# Coalescence and translation: A language model for population genetics

**DOI:** 10.1073/pnas.2518956123

**Published:** 2026-04-10

**Authors:** Kevin Korfmann, Nathaniel S. Pope, Melinda Meleghy, Aurélien Tellier, Andrew D. Kern

**Affiliations:** ^a^Institute of Ecology and Evolution, University of Oregon, Eugene, OR 97403; ^b^Department of Biology, University of Pennsylvania, Philadelphia, PA 19104; ^c^Department of Life Science Systems, Technical University of Munich, Freising 85354, Germany

**Keywords:** language models, coalescent theory, uncertainty, stdpopsim, simulation-based inference

## Abstract

Probabilistic models such as the sequentially Markovian coalescent have long provided a powerful framework for population genetic inference, enabling reconstruction of demographic history and ancestral relationships from genomic data. However, these methods are inherently specialized, relying on predefined assumptions and/or limited scalability. Recent advances in simulation and deep learning provide an alternative approach: learning directly to generalize from synthetic genetic data to infer hidden evolutionary processes. Here we reframe the inference of coalescence times as a problem of translation between two biological languages: the sparse, observable patterns of mutation along the genome and the unobservable ancestral recombination graph that gave rise to them. Inspired by large language models, we develop cxt, a decoder-only transformer that autoregressively predicts coalescent events conditioned on local mutational context. We show that cxt performs competitively with state-of-the-art Markov Chain Monte Carlo-based likelihood models across a broad range of demographic scenarios, matching their accuracy in-distribution and approaching it in out-of-distribution, with the potential for improvement via fine-tuning. Trained on simulations spanning the stdpopsim catalog [J. R. Adrion *et al*., *eLife* **9**, e54967 (2020); M. E. Lauterbur *et al*., *eLife* **12**, e84874 (2023); G. Gower *et al*., Accessible, realistic genome simulation with selection using stdpopsim. bioRxiv [Preprint] (2025). https://doi.org/10.1101/2025.03.23.644823.], the model generalizes robustly and enables efficient inference at scale, producing over a million coalescence predictions in minutes. cxt also produces well-calibrated approximate posteriors, enabling principled uncertainty quantification. We apply cxt to population genomic data from both humans and mosquitoes, highlighting the model’s ability to deal with the complexities of empirical data.

## Significance

cxt is a language model for population genetics which introduces next-coalescence prediction as translation from observed mutations to coalescence times by modeling the coalescent with recombination as a conditional stochastic process. It learns implicit priors from stdpopsim and generalizes across both known and novel demographies. cxt generates millions of the most recent common ancestor estimates in minutes and samples well-calibrated posteriors for uncertainty quantification. A simple post hoc correction aligns predicted diversity with the species mutation rate, ensuring robustness to novel evolutionary scenarios.

The stochastic process of genetic lineages tracing back to common ancestors over time gives rise to a complex structure known as the Ancestral Recombination Graph (ARG), with branches that merge through coalescence or divide through recombination (1–4). In the absence of mutations, these branches remain entirely concealed. However, mutations along the branches of the genealogy offer only a noisy, indirect window into this hidden ARG, carrying partial information about topology and the timing of events. Currently the field of population genetics is undergoing a revolution in our ability to infer the ARG that underlies a sample, and over the past few years we have obtained our first glimpses into reconstructions of whole genome genealogies (5–8).

Inferring the ARG (or approximations of it) has long been a central goal in population genetics. One of the earliest attempts was by Griffiths and Marjoram (9), who used Markov chain simulations of the coalescent process for ancestral inference. Subsequent work conceptualizing recombination as a point process along the genome (10), shifted focus toward emphasizing the spatial aspects of recombination, and laid the groundwork for more efficient approximations of the coalescent with recombination such as the sequentially Markovian coalescent (SMC) (11). The SMC approximation has been pivotal in the development of numerous inference methods, beginning with approaches requiring only a single diploid genome to investigate demographic changes, such as PSMC (12) (which effectively infers an ARG for two haploid samples). Since then, SMC-type methods have been extended in numerous ways including: to multiple genomes (13, 14), integration of site-frequency spectrum information (15), the inclusion of species-specific life-history traits (16–18), as well as algorithms that more efficiently scale as a function of sample size (19–21).

While the SMC approximation enables tractable likelihood-based inference for a restricted class of models, it becomes increasingly difficult to extend when key evolutionary processes, such as population structure, selection, or complex demographic events are introduced. Simulation-based inference (SBI) offers a flexible alternative by bypassing explicit likelihoods and instead learning mappings from data to model parameters directly from simulated datasets. Approximate Bayesian computation (ABC) is a widely used SBI framework in population genetics (22), and has been successfully applied to a range of inference problems, including demographic history reconstruction (23, 24).

However, classical ABC methods rely on reducing data to low-dimensional summary statistics in order to mitigate the curse of dimensionality, often discarding information relevant to inference in the process. This limitation is particularly acute for sequence-based problems involving recombination, where genealogical information is distributed across long, high-dimensional haplotypic sequences. In such settings, exemplified by PSMC-like models, information loss during data compression can substantially limit predictive accuracy and robustness, motivating the development of inference approaches that operate directly on high-dimensional sequence data (see, however, ref. 25).

A powerful alternative to standard ABC is to utilize deep learning (DL) for simulation-based inference, which has proven broadly effective across diverse domains (26). In population genetics, DL-based methods have been successfully applied to a wide range of model-based inference tasks (27), leveraging standard neural network architectures including fully connected networks (28, 29), convolutional neural networks (30–37), recurrent neural networks (38), and variational autoencoders (39). However, most existing DL approaches in population genetics are trained for narrow, task-specific objectives, such as estimating individual demographic parameters, and often fail to generalize beyond the distribution of their training simulations. While domain adaptation strategies can partially mitigate this limitation (40), they do not fundamentally address the challenge of learning transferable representations of the underlying evolutionary process.

More recently, transformer-based language models have been introduced that enhance prediction capabilities through the learning of stochastic processes, rather than scenario-constrained parameter estimation (37). Inspired by pretrained generative models such as GPT (41), we leverage this paradigm with a small model (10 to 20M parameters) that translates mutational patterns across chromosomes to coalescent time estimates, opening the door to a generalizable approach: pretraining on diverse coalescent simulations, followed by fine-tuning for specific evolutionary tasks if necessary.

In this work, we introduce cxt, a language model for coalescent estimation to our knowledge. cxt is a decoder-only transformer model inspired by GPT-2 and designed for pairwise time to the most recent common ancestor (TMRCA) prediction—a core component of genealogical inference. In contrast to DNA language models such as GPN-MSA or Evo 2 (42, 43) that are pretrained on the task of masked base prediction from a collection of sequenced genomes, our pretext task leverages synthetic data simulated under explicit population genetic models to predict the *next* pairwise TMRCA along a sequence. Rather than modeling DNA sequences itself, cxt aims to recover latent genealogical structure, providing a flexible and generalizable model for downstream inference. To enable this inference, we define a task analogous to next-token prediction, which we term next-coalescence prediction: The model autoregressively predicts the next pairwise coalescence time conditioned on local mutation densities within a fixed context window. While this does not reconstruct full genealogical topologies, it provides a structured translation from observed mutation patterns to pairwise coalescence times. Conceptually, cxt is a close neural analog of SMC++: Prediction is centered on a pivot (distinguished) pair, with information shared across haplotypes through the SFS.

To evaluate this approach, we focused on three criteria: 1) **accuracy**, performance comparable to or better than recent theory-driven methods such as Singer and SMC++; 2) **robustness**, particularly under model misspecification and out-of-distribution scenarios; and 3) **capacity**, the ability to generalize across a diverse range of simulated evolutionary scenarios.

Using extensive simulations and applications to human and mosquito genomic variation data, we show that cxt provides competitive performance for inferring local coalescence times across recombining chromosomes, with efficient parallel GPU inference. The generative nature of cxt enables rapid sampling of an approximate posterior over TMRCA trajectories, providing uncertainty estimates that are well calibrated. Across a wide range of models, including nearly the full stdpopsim catalog, cxt maintains reasonable out-of-sample performance, though with some loss in accuracy relative to likelihood-based methods, and can be further improved via targeted fine-tuning. Finally, we apply cxt to large-scale human and *Anopheles* population genomic data (44–47), recovering known coalescent-time outliers in humans and time-resolved signals consistent with insecticide resistance dynamics in mosquitoes.

## Results

**Fig. 1.**
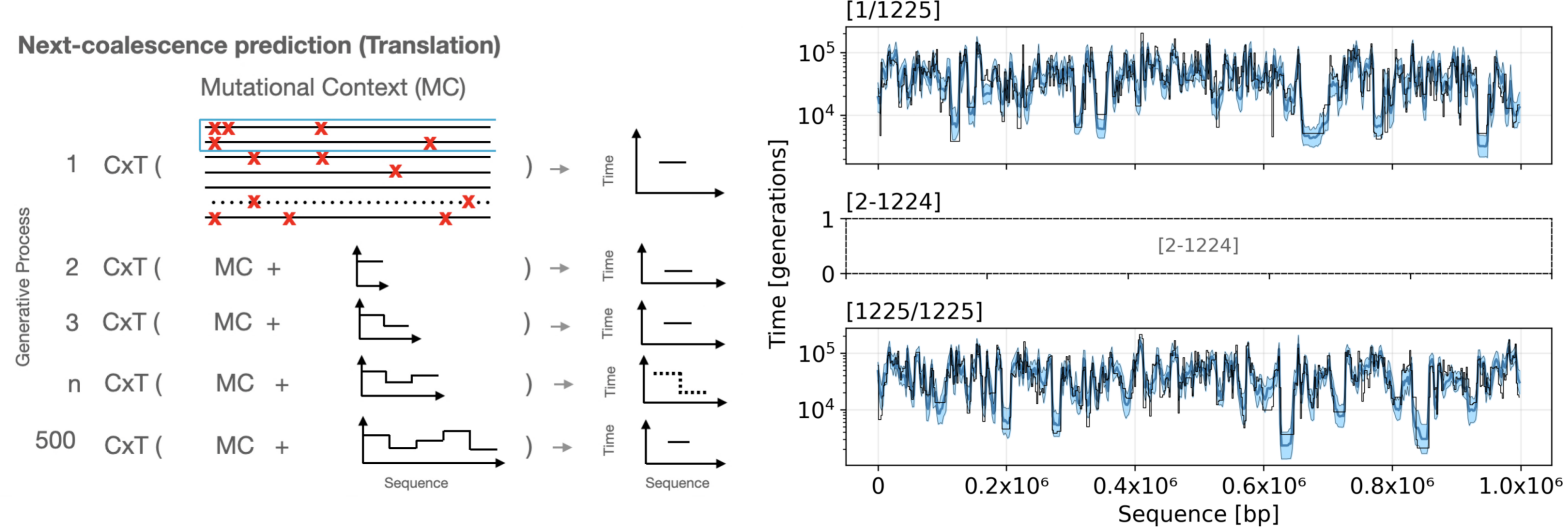
cxt introduces the notation of *next-coalescence prediction* (*Left*). cxt is a language model that conditions on a chosen “pivot” haplotype pair and predicts the pair’s time to the most recent common ancestor (TMRCA) for each window. cxt ingests mutational tensors constructed using the pivot pair and site–frequency–spectrum (SFS) values computed in windows across a focal region. The model works autoregressively: after each window is predicted, that estimate is appended to the context and supplied to the next step, yielding a step-wise reconstruction of the entire pairwise coalescent history. Because every haplotype pair can be processed in parallel, the approach scales efficiently with available GPU memory. In the example application (*Right*), all $\left(\begin{array}{c} 50\\ 2 \end{array}\right)$ pairwise coalescence curves for a sample of 50 haplotypes were inferred simultaneously in under five minutes on a single NVIDIA A100 GPU. True TMRCAs are plotted in black, individual replicate predictions in light blue, and the prediction mean in dark blue.

We begin by presenting a conceptual schematic that adapts the general language-modeling paradigm to population genetics, and draw parallels with SMC-based coalescent modeling to define our next-coalescence prediction task (Fig. 1). We then benchmark cxt’s accuracy against current fast inference methods, specifically Singer and SMC++ (8, 15). Next, we evaluate the model’s cross-scenario generalization capabilities, training and evaluating using the nearly complete stdpopsim v0.2 and v0.3 catalogs (48–50) across diverse species, demographic scenarios, and genetic maps (excluding only bacteria and two species with extreme recombination rates that preclude large-scale simulation and three models with ancient samples). We assess the global accuracy of TMRCA prediction by comparing true and estimated coalescence rates across simulations. Finally, we apply cxt to real genomic data, estimating coalescent times across two chromosomes from the 1000 Genomes Project, using pairs of haploid sequences of British ancestry as our focal samples and chromosome 2L of *Anopheles* from samples taken from Cameroon, Mali, Burkina Faso, Ghana and Uganda.

### Fast and Accurate Inference.

We developed cxt, a decoder-only transformer architecture adapted for the task of inferring local coalescence times from mutation patterns across recombining genomes. Unlike language models that embed discrete tokens, cxt projects continuous mutation densities into a latent space using feed-forward networks, followed by attention-based contextualization. Rotary positional embeddings encode physical distance along the genome, allowing the model to capture locality in genealogical relationships. Trained using a next-coalescence prediction objective, cxt captures the stochasticity of evolutionary processes while achieving rapid and accurate inference.

We show a descriptive schematic of the model in the *Left* panel of Fig. 1. cxt follows a general language-modeling paradigm in which the model predicts a discretized coalescent time for each genomic window based on preceding predictions and the local mutational context. Specifically, we divide the genome into fixed-size positional windows and discretize coalescent times into bins on a logarithmic scale. Given a sequence of ground truth discretized pairwise TMRCA values $E=(e_{1},e_{2},\cdots ,e_{n})$, the model autoregressively learns the conditional distribution:

$$P\left(E|\text{MC}\right)=\prod_{i=1}^{n}P\left(e_{i}|{\hat{e}}_{<i},\text{MC}\right),$$

where ${\hat{e}}_{<i}$ represents the model’s stochastic predictions for the preceding windows and MC denotes the mutational context (the observed data) across all windows. Concretely, the mutational context in a given window is the site frequency spectrum calculated across all samples, partitioned into the contributions from sites that are heterozygous and sites that are homozygous in the pivot pair.

At each genomic window *i*, the model outputs a probability distribution over discretized TMRCA bins:

$${\hat{e}}_{i}∼P_{\theta }\left(e_{i}|{\hat{e}}_{<i},\text{MC}\right),$$

with $P_{\theta }$ parameterized by a neural network with weights *θ*. The training objective is to maximize the log probability of the true sequence given the (preceding) predicted sequence:

$$L\left(\theta \right)=\sum_{i=1}^{n}\log P_{\theta }\left(e_{i}|{\hat{e}}_{<i},\text{MC}\right),$$

where $e_{i}$ denotes the ground truth label in window *i*, and ${\hat{e}}_{<i}$ are the model’s own predictions up to (but not including) window *i*. Predictions are then centered around an intercept (log expected total diversity), which is sampled from the posterior distribution conditional on observed total diversity assuming a Poisson model of mutation. This objective encourages the model to learn a mapping from local mutation patterns to genealogical structure, enabling accurate sequential inference of coalescent events across the genome.

The *Right* panel of Fig. 1 illustrates cxt on a constant-size scenario ($N_{e}=2\times 10^{4}$, roughly equal mutation and recombination rates; see Datasets). All $\left(\begin{array}{c} 50\\ 2 \end{array}\right)$ pivot pairs of 50 haploid chromosomes are inferred in parallel; for each pair the generative process is repeated 15 times, yielding samples from an approximate posterior over pairwise coalescence times along the sequence (where the prior is implicit via the simulated training set; see Calibration). Averaging across replicates produces stable estimates. In this well-specified setting cxt is highly accurate, and inference for all pairs completes in approximately five minutes on a single NVIDIA A100 GPU.

### Benchmark Comparisons.

To evaluate cxt’s performance, we benchmarked its accuracy against two approaches Singer and SMC++ (8, 15), selected for their accuracy and broad adoption, respectively.

**Fig. 2.**
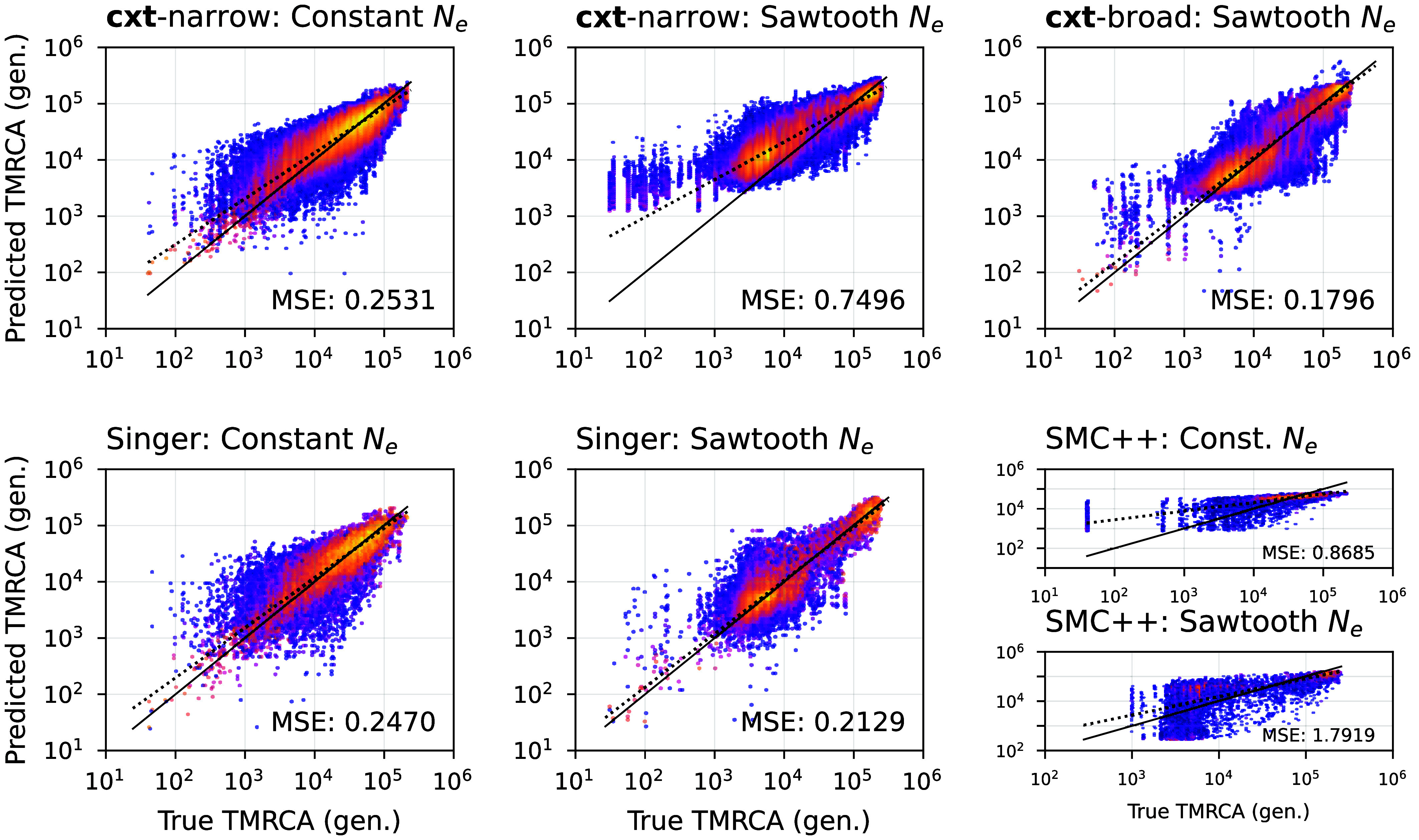
True versus predicted coalescence times for three inference approaches across two demographic scenarios: a constant population size and a fluctuating “sawtooth” demography. *Top* row: cxt-narrow evaluated on the constant-size scenario (*Left*) and on the sawtooth scenario (*Middle*), followed by cxt-broad evaluated on the sawtooth scenario (*Right*). The broad model—trained on a wider range of demographic histories—achieves substantially improved accuracy. *Bottom* row: Singer+Polegon evaluated on the constant-size (*Left*) and sawtooth (*Middle*) scenarios. The two rightmost panels show SMC++ applied to the constant-size and sawtooth scenarios, respectively. Although SMC++ is primarily designed for population-size inference, we include its decoding-based coalescence-time estimates for completeness. Mean squared errors (MSE) for each panel are reported within the plots.

We first consider the performance of our “narrow model,” a cxt model trained on a constant-sized population ($N_{e}=2\times 10^{4}$) with equal mutation and recombination rates (see Datasets). In Fig. 2, we aim to demonstrate two main results. First, we show the performance of the narrow model (**cxt**-narrow, *Top-Left*) on its original training domain, constant population size with roughly equal mutation and recombination rates, which yields a mean squared error (MSE) of 0.2531. As expected, **cxt**-narrow’s predictive accuracy declines when evaluated outside this domain. The *Top-Middle* panel shows results under a fluctuating population-size model (the sawtooth scenario; MSE 0.7496); thus, model misspecification degrades performance. We can potentially mitigate this behavior by training cxt on a wider range of parameters and demographic histories. To do so, we introduce a “broad model,” **cxt**-broad (*Top-Right*), trained on a substantially wider range of scenarios (see Datasets). **cxt**-broad, because it can accommodate demographic variation, achieves improved accuracy on the sawtooth model (MSE 0.1796).

Singer, combined with its dating-refinement algorithm Polegon (**Singer+Polegon**), attains comparable accuracy on the constant-size scenario (Fig. 2*Bottom-Left*; MSE 0.2470) and slightly reduced accuracy under the sawtooth model (MSE 0.2129). **SMC++**, which is primarily designed for demographic history inference with TMRCA decoding provided as part of its composite-likelihood framework, yields higher MSE values for these simulations (0.8685 and 1.7919), reflecting its emphasis on population-size reconstruction rather than fine-scale local genealogy prediction.

Because cxt is trained at a fixed sample size, users with larger samples can subsample, while for smaller samples we provide a lightweight adapter that fine-tunes the pretrained model to the target cohort size. *SI Appendix*, Fig. S4 shows adapter results transferring from N=25 to N=5 diploids on both the constant-size and sawtooth scenarios.

Our results demonstrate that the language-modeling approach implemented in cxt is competitive with the most recent theory-driven methods, whether ARG-based (as in Singer) or SMC-based (as in SMC++), in these well-specified settings. A full summary of error estimates is provided in *SI Appendix*, Table S6 and Fig. 2.

### Computational Efficiency and Scaling.

We compared wall-clock runtimes of cxt with SMC++ and Singer across increasing numbers of inferred pairwise coalescence trajectories (*SI Appendix*, Fig. S6). Overall, cxt exhibits approximately linear scaling in the number of inferred pairs and near-linear speedups with additional GPUs, while likelihood- and MCMC-based methods incur substantially higher and more parameter-sensitive computational costs. Detailed runtime breakdowns and scaling analyses are provided in *SI Appendix*.

### Toward a Generalizable Deep Learning Model.

In the previous section, we focused primarily on the setting in which cxt is trained on a single demographic model (cxt-narrow), thereby learning directly from simulated data, while also providing a preview of cxt-broad’s potential. The central advantage of a language-modeling approach is its capacity, through many learnable parameters, to move beyond narrowly specified generative assumptions by conditioning its behavior on a rich set of mutational and genealogical contexts. In this sense, cxt functions as a flexible conditional density estimator: For a given context, it produces a corresponding predictive distribution. We assess this directly by comparing inferred marginal coalescence-time distributions across species.

In *SI Appendix*, Fig. S7, we report inferred coalescence time distributions across nearly all stdpopsim v0.2 species. For each species, we simulate under its published demographic model when available, and otherwise assume a constant population size. Results from simulations using fine-scale recombination maps are given separately in *SI Appendix*, Fig. S8. All results shown here are based on the cxt-broad model (see Datasets), which was trained across the full set of scenarios. Importantly, the evaluations in *SI Appendix*, Figs. S7 and S8 are performed on newly generated simulations that were not included in training, eliminating any opportunity for data leakage. Across nearly all species, the inferred distributions (blue) closely match the true distributions (black).

To promote generalization across demographic and rate regimes, we made a deliberate design choice during training: We keep the window size fixed at 2 kb while varying $N_{e}$ over several orders of magnitude. In principle, one might expect the window size to scale with the effective population size, since the expected numbers of mutations and recombination events per window scale with $N_{e}$. However, by intentionally not adapting the window size, the model encounters windows that can be mutation-dense or mutation-sparse depending on the underlying $N_{e}$. This encourages the model to learn *relative* changes in local coalescent structure rather than relying on absolute mutation counts; any global offset can be corrected during inference by calibrating to the mutation rate.

A related consideration is how well the mean coalescent time within a window can be estimated. As the window size increases, estimation becomes easier: mutation counts rise, diversity increases, and sampling noise decreases. However, larger windows also average over spatial variation in genealogical structure, attenuating signals of population-size change, population structure, and other localized effects.

We illustrate this trade-off in *SI Appendix*, Fig. S5. Using 2 kb windows (*Left*; MSE 0.1096) predicted coalescence times closely track the truth for the stdpopsim *AnoGam* model. In contrast, using much smaller 0.2 kb windows (*Middle*; MSE 0.5727) substantially increases difficulty: More frequent recombination induces greater heterogeneity among local genealogies, while fewer mutations per window increase variance in the observed signal. Despite the broad model’s reasonable performance in this setting, we further fine-tune it on stdpopsim species with $N_{e}>10^{5}$ (*SI Appendix*, Fig. S5, *Right*). This yields the broad (w200) model and further reduces prediction error.

### Out-of-Sample Tests for the Broad Model.

During this project, stdpopsim v0.3 was released (49), expanding the catalog to include additional species (*Sus scrofa, Rattus norvegicus, Phocoena sinus, Mus musculus*, and *Gorilla gorilla*). We leveraged this release to assess the generalization ability of our broad model, testing performance *without* retraining. Because these species were not present in stdpopsim v0.2, they constitute genuinely out-of-sample test cases for the broad model.

The v0.3 release includes species spanning a wide range of effective population sizes, from very large populations, such as *S. scrofa* ($N_{e}=270,000$) and *R. norvegicus* ($N_{e}=124,000$), to much smaller populations, such as *P. sinus* ($N_{e}=3,500$). Most of these new species also exhibit recombination rates that exceed mutation rates. For example, in *P. sinus*, we have $m=5.83\times 10^{-9}$ and $r=1\times 10^{-8}$ per bp per generation (r/m=1.715). A notable exception is *G. gorilla* for which $r=1.193\times 10^{-8}$ and $m=1.235\times 10^{-8}$, yielding r/m=0.966.

**Fig. 3.**
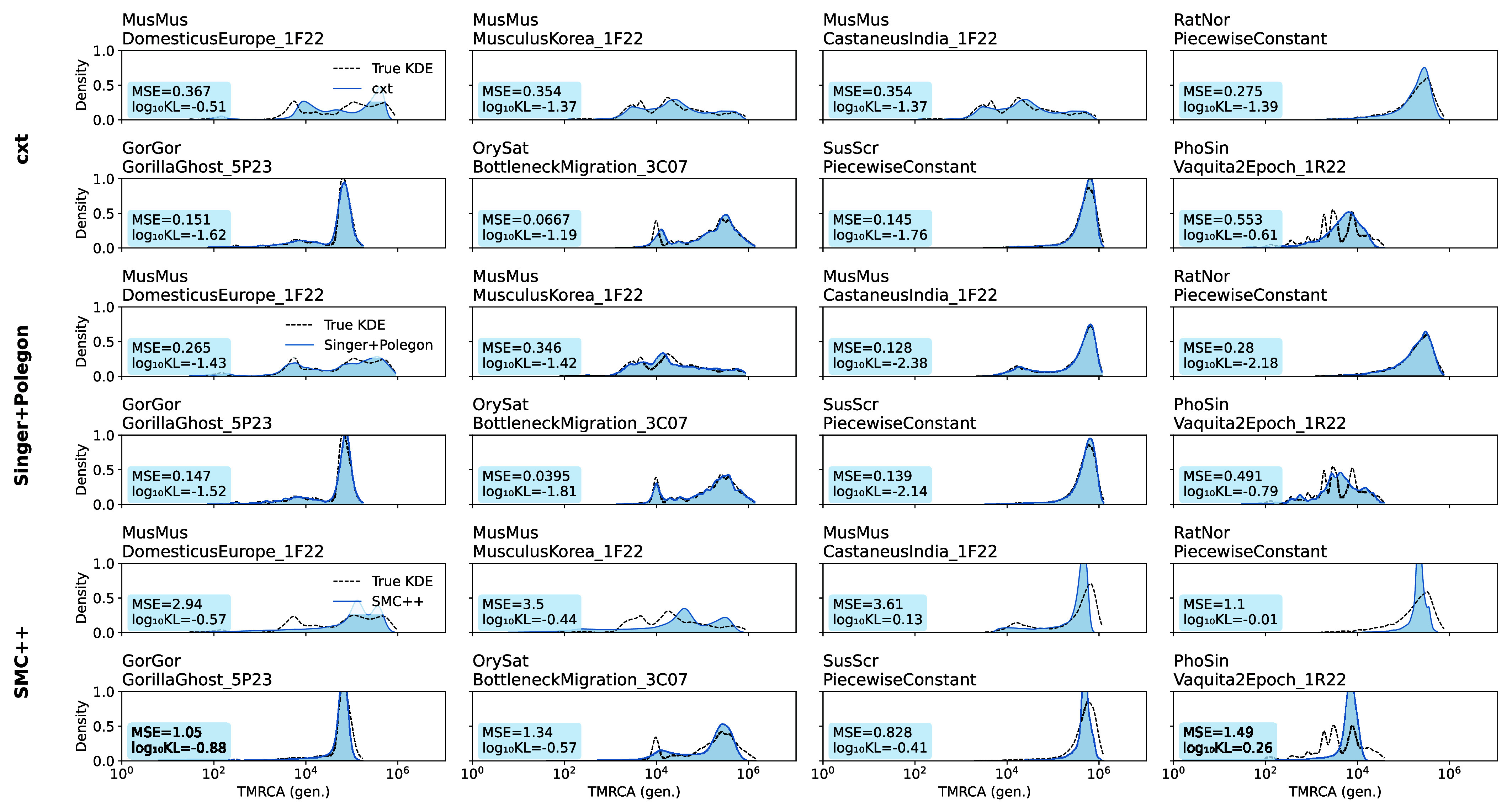
Out-of-sample evaluation of the broad model on stdpopsim v0.3. Each panel shows inferred marginal coalescence distributions (dashed) against true distributions (shaded). We simulated data from the novel species / demographic histories added in stdpopsim v0.3, aggregating pairwise inferences into kernel density plots. All results are from simulations previously unseen during training (*Top* Rows: cxt; *Middle* Rows: Singer+Polegon; and *Bottom* Rows: SMC++).

Results from this out-of-sample evaluation are shown in Fig. 3. Notably, cxt generalizes well, even when applied to simulations generated under previously unseen regimes of recombination and mutation rates, population sizes and demographic structure. Across these v0.3 species, cxt generalizes reasonably well, although Singer+Polegon achieves lower error in most scenarios, in some cases by a substantial margin. Both methods substantially outperform the SMC++ decodings.

### Generalization Across Parameter Regimes.

A central goal of this project was to build a model that spans the diversity of scenarios represented in stdpopsim, motivated by the idea that as stdpopsim expands, we can incrementally incorporate new species into training. However, when applying cxt to data from a new species, with potentially different mutation and recombination rates, effective population size or population structure, we expect inference accuracy to depend on how similar that species is to the regimes represented in the training data.

We assessed cxt’s ability to interpolate and extrapolate beyond the training distribution by evaluating prediction accuracy across a grid of mutation and recombination rates at fixed $N_{e}=20,000$ (*SI Appendix*, Fig. S10). The grid is chosen to span most stdpopsim species; MSE and KL divergence heatmaps show that, as expected, error is highest in the low-mutation, high-recombination regime where the signal-to-noise ratio is least favorable. Representative TMRCA predictions from the four corners of the grid (*SI Appendix*, Fig. S11) confirm that cxt generalizes beyond the parameter regions represented in training.

### Calibration of Approximate Posteriors.

In addition to point estimates, cxt produces approximate posterior samples for local TMRCA, enabling uncertainty quantification. We evaluated the frequentist calibration and consistency of these posteriors across multiple demographic scenarios and mutation-rate regimes, including out-of-training species. Overall, posterior intervals show near-nominal empirical coverage and contract appropriately with increasing mutational information, with deviations confined to boundary windows where context is truncated. Detailed calibration analyses are provided in *SI Appendix*, Figs. S2, S3, and S9.

### Demography Estimation Through Coalescence Rates.

**Fig. 4.**
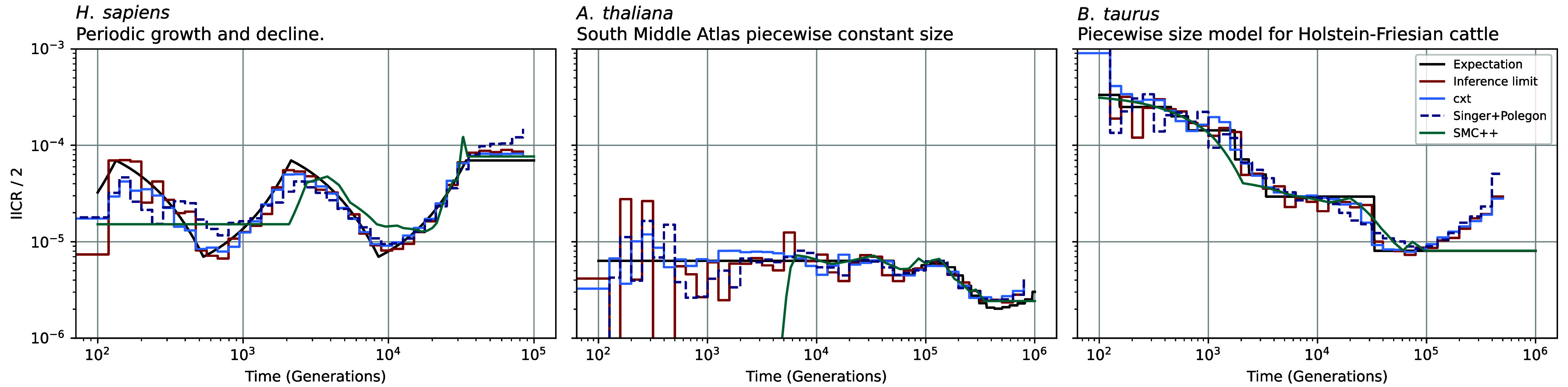
Inverse-instantaneous coalescence rate calculation for the piecewise-constant demography of *H. sapiens* (*Left*), *A. thaliana* (*Middle*), and *B. taurus* (*Right*). The inference of pairwise-coalescence events leads to the implicit inference of demography estimates through the marginal coalescence distribution assuming coalescence occurs as a Poisson process (*Materials and Methods*). For each scenario 10 Mb and 25 diploid samples have been used to achieve resolution throughout the specified time-windows.

Estimating pairwise coalescence rates provides a direct window into changes in effective population size through time, perhaps most famously exemplified by the original PSMC model of Li and Durbin (12). To evaluate our ability to recover effective population size trajectories from cxt predictions, we simulated data under demographic models for *Homo sapiens*, *Bos taurus*, and *Arabidopsis thaliana*. From these simulations, we estimated pairwise coalescence-time distributions and converted them into coalescence-rate curves, which we then mapped to estimates of historical effective population size (Fig. 4; see *Materials and Methods*).

Fig. 4 shows five curves. The black *expectation* line reflects the true coalescence rate implied by the generative model. We also report an *inference limit* in red, constructed by sampling the true TMRCAs every 2 kb across the sequence and then estimating coalescence rates using the same procedure applied to cxt, Singer, and SMC++, thereby providing an upper bound under matched discretization. The remaining curves show the corresponding estimates from cxt, Singer, and SMC++.

Overall, effective population size trajectories inferred from cxt and Singer closely track the truth at a genome-wide scale capturing the major epochs of growth/bottlenecks, whereas SMC++ shows larger deviations at recent times; this gap may reflect limited information in the current setting and could plausibly be reduced by incorporating additional data.

We also inferred the cross-coalescence rate (i.e., the rate of coalescence between lineages drawn from different demes) under the two-population OutOfAfrica_2T12 model from stdpopsim. Generally, cross-coalescence curves are informative about the timing of divergence and subsequent gene flow between populations within a species. *SI Appendix*, Fig. S12 summarizes these results. The *Left* panel shows the cross-population rate estimated from one African and one European haplotype, whereas the *Middle* and *Right* panels show the within-population rates for African and European samples, respectively. The cross-population curve (*Left*) closely follows the expected trajectory. In contrast, the within-population curves exhibit a modest deviation for cxt.

A likely explanation is the way structured models were represented during training: For each simulated instance, 50 samples were drawn with random population assignments. As a result, training examples rarely contained only a single population, which may bias within-population rate estimation. This could potentially be mitigated by modifying the training scheme for structured models, for example by explicitly including single-deme sampling configurations.

These demographic inferences are based on 10 Mb of simulated sequence (Fig. 4 and *SI Appendix*, Fig. S12); because shorter sequences truncate the longest tracts of recent ancestry, increasing variance in recent-time rate estimates, we expect substantially improved accuracy at full chromosome lengths. Overall, cxt’s local TMRCA predictions aggregate into accurate genome-scale demographic summaries.

### Application to Human 1000 Genomes and Ag1000G Mosquito Data.

Having established the accuracy and robustness of cxt across a broad range of simulated scenarios, we next apply it to empirical genomic data: human variation from the 1000 Genomes Project (44) and mosquito genomes from the Ag1000G consortium (46).

We begin with the human dataset, revisiting the canonical selective sweep at *LCT* as a validation benchmark, and then turning to more recently dated regions of the *HLA* locus. In the latter case, our inferred TMRCA patterns are broadly consistent with previously reported old genealogical ages, though we infer slightly younger times than Singer+Polegon.

We then analyze the insecticide-resistance loci *Rdl* in *Anopheles gambiae*. This locus has been highlighted using summary-statistic approaches, including Garud’s $H_{1}/H_{12}$, but a clear time-resolved interpretation of the underlying sweep dynamics has remained elusive. Using cxt, we estimate fine-scale coalescent times across five African populations (Burkina Faso, Mali, Cameroon, Ghana, and Uganda) while addressing three technical challenges characteristic of Ag1000G data, and we compare our mosquito estimates to Singer+Polegon:

1. **Large effective population sizes**, which yield deep genealogies and correspondingly large scaled mutation and recombination rates;
2. **Small sample sizes** in Ghana (<25 diploid individuals); and
3. **Substantial missing-data variation** across genomic regions

To begin this initial validation in humans, we selected 50 individuals of British descent (GBR), and estimated coalescent times for 25 focal pairs, holding out the remaining n=48 samples as the AFS input to cxt.

**Fig. 5.**
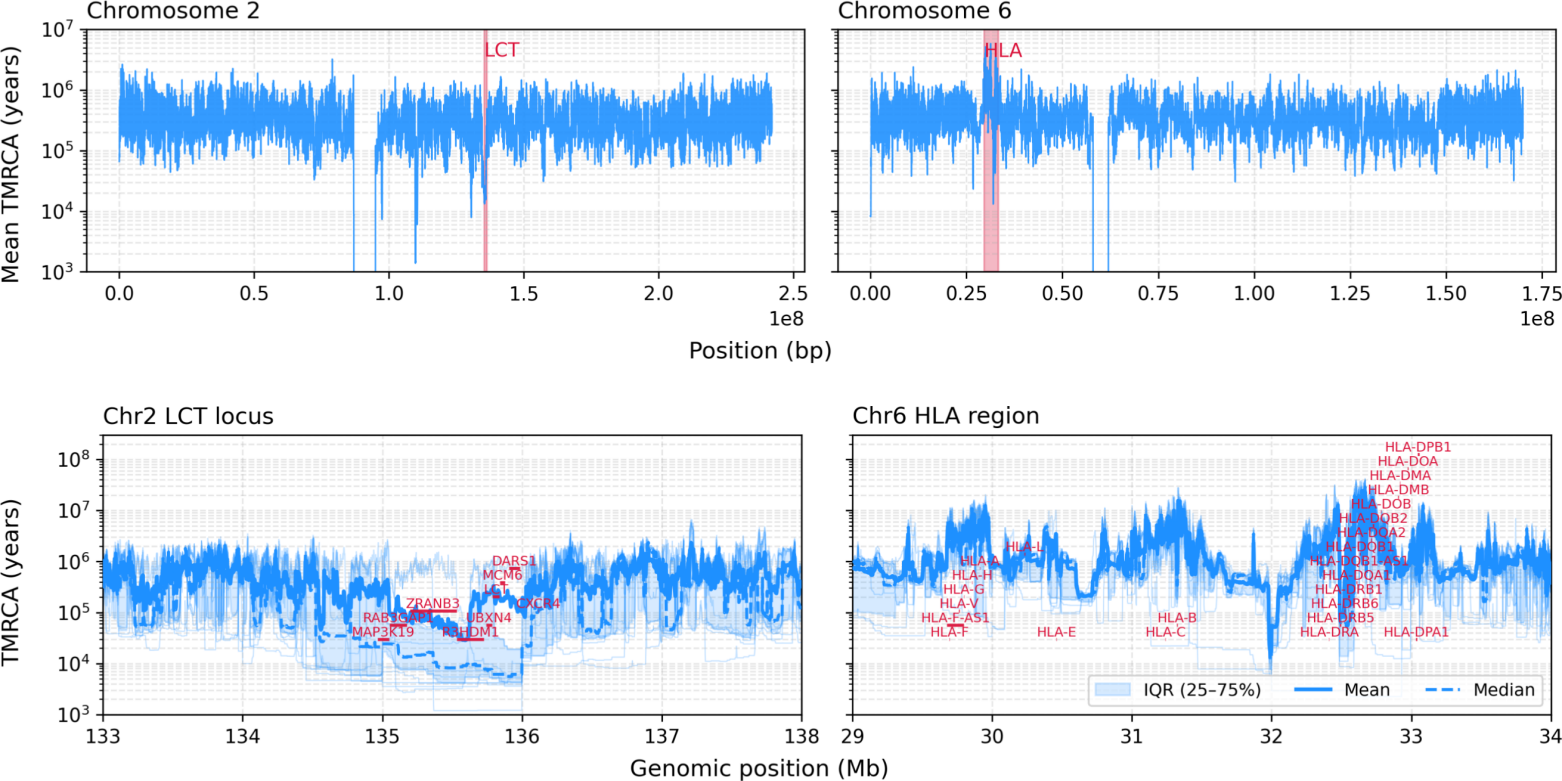
Inference of coalescent times from 25 focal pairs of British individuals of the 1000 Genomes project. The *Top* panel shows chromosome-wide TMRCA landscapes for chromosomes 2 (*Left*) and 6 (*Right*). The *Bottom* panel zooms into the LCT region on chromosome 2 (*Left*) and the HLA region on chromosome 6 (*Right*). Each pivot pair has been inferred 15 times to get a reliable mean, median and variance estimates. Additionally, light blue lines show the respective inferences of the 25 pairs.

We choose 25 focal pairs because this sample size allows all chromosome-wide comparisons to fit in a single batch on an A100 GPU, enabling rapid whole-chromosome inference. The *Top* panels of Fig. 5 show chromosome-wide TMRCA landscapes for chromosomes 2 and 6. As an additional validation, we compared inferred TMRCAs against the number of recombination events predicted by cxt (*SI Appendix*, Fig. S15), recovering the expected inverse relationship between genealogical age and segment length.

The *Bottom* panels in Fig. 5 zoom in on two canonical regions: the *LCT* locus on chromosome 2 and the *HLA* region on chromosome 6. At *LCT*, we recover markedly reduced coalescent times as expected under the well-characterized recent selective sweep at this locus. cxt captures this local trough cleanly. Around *LCT*, estimated coalescent times slightly above 10 kya are consistent with the origin of the sweeping haplotype, and the variation across focal pairs highlights local heterogeneity.

The *HLA* region provides the complementary pattern: exceptionally deep genealogical structure shaped by long-term balancing selection. The extended peaks inferred by cxt (*Bottom-Right*) match long-standing expectations for this locus and support the conclusion that the model captures extreme TMRCA variation. Consistent with this, we replicate the multipeaked ancient structure reported by ref. 51, with several genes reaching TMRCAs on the order of tens of millions of years, one of the strongest known signatures of long-term balancing selection.

**Fig. 6.**
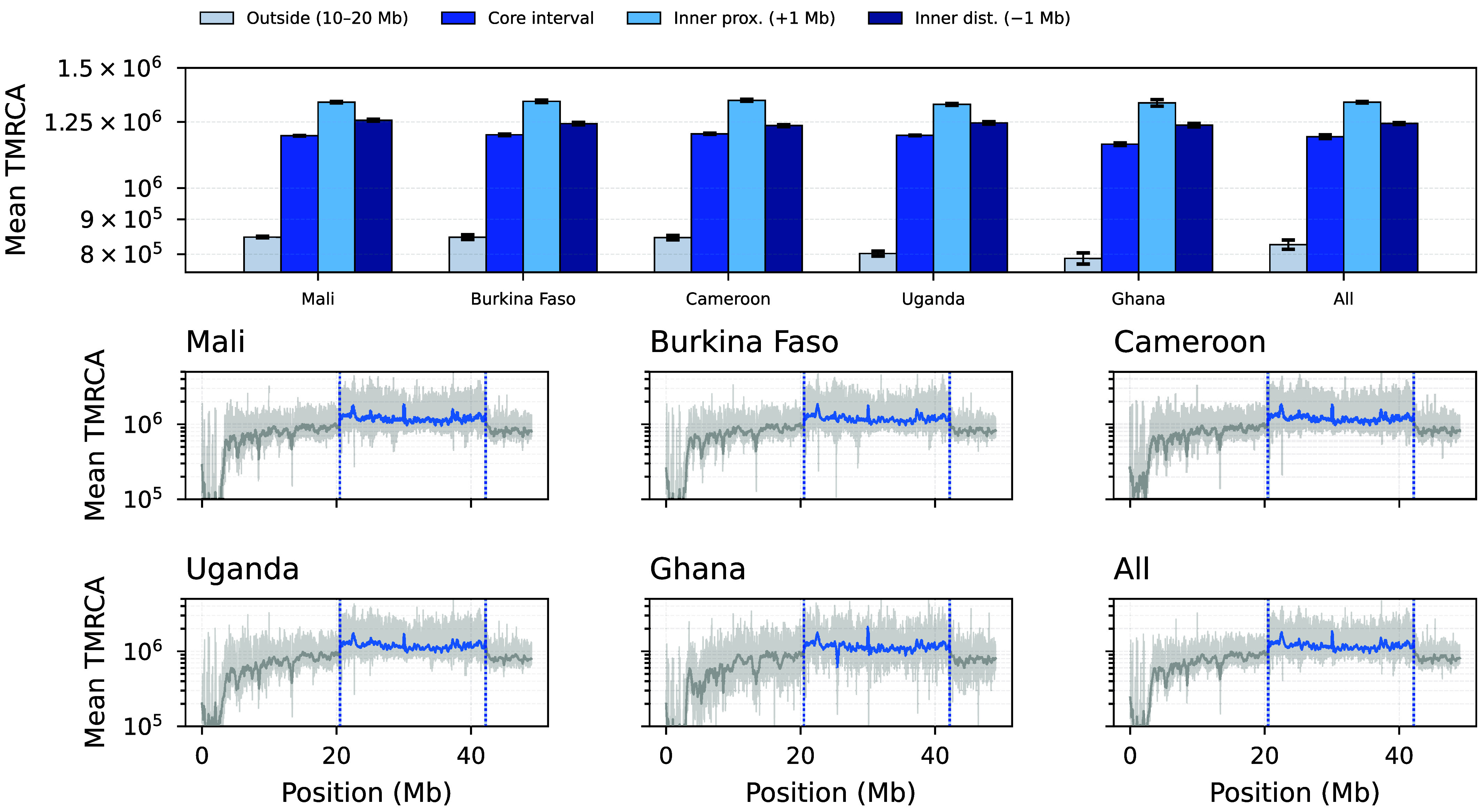
Coalescent-time structure across the *In(2L)a* inversion on chr2L. *Top*: mean TMRCA summaries for an outside background region (10 to 20 Mb), the full inversion core interval, and two interior 0.5 Mb windows positioned 1 Mb inside each breakpoint (mean ± s.e.m. across replicate trajectories; “All” aggregates populations). *Bottom*: genome-wide mean TMRCA curves shown per population and pooled (“All”), with vertical guides marking inversion boundaries, breakpoint-adjacent windows, and the interior sampling windows used for summary statistics.

To investigate selective dynamics at the *Rdl* locus across multiple *A. gambiae* populations, we constructed a comparison panel (*SI Appendix*, Fig. S14) showing coalescent-time landscapes from cxt, Singer+Polegon, and SMC++. The challenges outlined above are particularly acute here; high $N_{e}$-scaled rates substantially slow MCMC-based approaches such as Singer+Polegon. For readability, we show only cxt results in the main text (Fig. 6; see also *SI Appendix*, Fig. S13); full method comparisons appear in *SI Appendix*, Fig. S14.

To address these issues, we fine-tuned cxt on a filtered stdpopsim training set restricted to $N_{e}>10^{5}$, reduced the window size ten-fold to 200 bp, and performed inference in 100 kb blocks. We additionally incorporated Ag1000G missing-data masks during fine-tuning to improve robustness to region-specific missingness.

Finally, because the Ghana population does not meet the 25-diploid sample size used elsewhere, we trained a small adapter model to handle reduced sample size, while jointly accounting for large $N_{e}$ and missing-data patterns.

The Singer+Polegon method was also run on the same samples. We tried two approaches to using Singer+Polegon: one with the “natural” parameterization of mutation and recombination rates, which for *Anopheles* yields a ratio of recombination:mutation >>1, and a second where we held this ratio fixed at 1 (equivalent to the human parameterization). The former, “natural” parameterization yielded poor fits to observed diversity, particularly the marginal site frequency spectrum, thus the results presented here do not use mutation and recombination rates that reflect what is known about the *Anopheles* genome, although the resulting fits are superior with respect to matching observed diversity patterns.

Both cxt and Singer+Polegon successfully recover the pronounced reduction in TMRCA at *Rdl*. Importantly, the depth of this trough is not uniform across populations: Some populations, such as Ghana, show a sharper, more localized depression, whereas Uganda shows no depression in TMRCA at all. This pattern parallels published differences in the frequency of *Rdl* insecticide-resistance alleles among these populations (52), supporting the interpretation that the inferred coalescent-time signal reflects geographically heterogeneous selection.

In comparing their outputs, we note a key difference in behavior in regions with missing data. Singer+Polegon consistently infers strong dips in the TMRCA in regions with missing data (*SI Appendix*, Fig. S14), presumably as a result of miscalibrated expectations of mutation rate in those regions. However, because cxt is explicitly trained to deal with missing data, no such artifacts are inferred.

SMC++ also detects a noticeable reduction in TMRCA around the *Rdl* sweep, but its blockwise representation limits localization. Even after scaling the output to match the true genomic span and calibrating genome-wide diversity to the cxt and Singer+Polegon panels, the inferred trough remains discretized and slightly shifted. This is expected: SMC++ fits a global demographic process using coarse blocks, so narrow sweeps can be detected but their genomic breakpoints are less precisely resolved.

#### Dating an inversion.

To characterize how the *In(2L)a* inversion shapes genealogical age across chr2L, we summarize mean TMRCA in i) an outside background region (10 to 20 Mb), ii) the full inversion core interval, and iii) two 0.5 Mb windows sampled *inside* the inversion, offset by 1 Mb from each breakpoint to avoid the immediate transition zone where mapping artifacts, missingness, and recombination suppression can confound interpretation. Averaged across populations, coalescent times are elevated inside the inversion relative to the outside background: outside (10 to 20 Mb) $=8.264\times 10^{5}\pm 2.875\times 10^{4}$ generations, core interval $=1.189\times 10^{6}\pm 1.674\times 10^{4}$, inner proximal (+1.0 Mb) $=1.336\times 10^{6}\pm 7.116\times 10^{3}$, and inner distal (−1.0 Mb) $=1.243\times 10^{6}\pm 8.939\times 10^{3}$ (mean ± s.d. across populations). This ordering (outside < core < inversion interior) is consistent with long-lived haplotype structure maintained within the inversion, as expected under reduced effective recombination between arrangements and/or long-term maintenance of both karyotypes. In practice, focusing on the inversion *interior* provides a conservative summary that reflects the inversion’s long-term genealogical signal while reducing sensitivity to breakpoint-adjacent artifacts.

## Discussion

In this study, we cast population-genetic inference as a language-modeling problem by training a modified GPT-2 model to learn the sequential structure of the coalescent with recombination as a conditional stochastic process. In spirit, the method is aligned with SMC-based approaches such as SMC++, but it replaces the explicit HMM likelihood, and its enforced first-order dependence, with attention-based sequence modeling. Modeling assumptions and priors are supplied implicitly through simulation during training, rather than specified analytically at inference time.

The core of cxt is an autoregressive conditional generator: Given observed mutations in a window, the model samples a discrete distribution over pairwise coalescence times and then extends predictions along the chromosome by conditioning on its own previously generated states. Repeating this procedure yields an approximate posterior over TMRCA trajectories that can be sampled efficiently in parallel. This approach differs fundamentally from prior deep learning methods such as ReLERNN (38) or CoalNN (37), which treat inference along the chromosome as a supervised regression task, and produce a single point estimate from sequence data. In contrast, cxt uses a language model to learn the underlying conditional stochastic process.

cxt produces genome-wide TMRCA estimates for a diploid individual in minutes on a single GPU, and it can alternatively focus on a specific region and decode many pairs locally. Comparisons to SMC++ and Singer+Polegon show that cxt consistently outperforms SMC++ in terms of accuracy of TMRCA inference (*SI Appendix*, Table S6), and performs competitively with the state-of-the-art ARG-based method Singer+Polegon, matching it in well-specified settings, though with higher error in some out-of-distribution scenarios. Singer+Polegon remains the more accurate choice when computational cost is not a constraint, but achieving competitive performance without explicit ARG modeling highlights the efficiency of the language-model approach. These computational and statistical properties motivate application to empirical datasets spanning both canonical and challenging inference regimes.

To assess the performance of cxt on empirical data, we applied it to two empirical systems with very different mutation-to-recombination regimes: i) a targeted validation in humans of the *HLA* and *LCT* regions of the genome, known coalescent time outliers, and ii) a chromosome-scale scan of chr2L in Anopheles, highlighting the extensibility of our model to settings with huge population sizes and pervasive missing data that challenge standard statistical estimation.

In humans, the *LCT* locus provides a canonical example of recent, strong, and geographically structured selection. Lactase persistence is driven primarily by regulatory variation upstream of *LCT*, including variants located in intronic sequence of the neighboring *MCM6* gene that modulate enhancer/promoter activity (53, 54). Population-genetic analyses indicate that derived lactase-persistence haplotypes rose rapidly in frequency over roughly the past 5,000 to 10,000 y, consistent with gene–culture coevolution in the context of dairying (55, 56). Our pairwise TMRCA estimates at *LCT* reflect this known history cleanly, recovering a pronounced local dip in coalescence times centered on the locus. In our sample, some pairs exhibit a TMRCA younger than 10,000 y, substantially younger than the average across the region, but we note that there is extensive variation across pairs that likely reflects that LCT arose from standing variation (57), and thus the locus harbors significant variability in the underlying genealogies.

In the *HLA* region, we observe exceptionally ancient separation times among alleles. For several genes, pairwise TMRCAs exceed commonly cited human–chimpanzee divergence times, consistent with trans-species polymorphism under long-term balancing selection. The *HLA* locus is among the most polymorphic regions of the human genome, likely driven to extreme variation by arms-race dynamics at the immune genes it encodes. Accordingly, there is abundant evidence that some *HLA* alleles have been maintained for tens of millions of years by balancing selection (58–60). In our sample of British individuals, the *HLA* region is a clear outlier in the chromosome-wide distribution of coalescence times (Fig. 5, *Top Right*). Across multiple genes, we observe pairwise TMRCAs exceeding 10 My. While surprisingly ancient, these estimates are nonetheless in agreement with recent population-genetic dating in ref. 51. Together, *LCT* and *HLA* provide complementary extremes, recent directional selection versus long-term balancing selection, allowing us to validate cxt across nearly the full dynamic range of human genealogical timescales, from thousands to tens of millions of years.

Unlike the human analyses, which primarily serve as validation against well-characterized loci, the Ag1000G application reveals previously unresolved spatial and temporal structure within ancient inversions and insecticide-resistance sweeps. These mosquito data also present a substantially more challenging setting for coalescent inference. In particular, the Ag1000G data combine very large effective population sizes, pervasive and spatially heterogeneous missingness, and uneven sample sizes across populations. Together, these factors challenge both diversity-based summaries and likelihood-based decoding, and provide a stringent test of whether cxt can be adapted to real data with nonideal sampling and observation patterns.

We highlight chromosome 2L which carries a number of known targets of insecticide resistance as well as a well studied inversion, *In(2L)a*. Considering first the chromosomal landscape of estimates (*SI Appendix*, Fig. S13, *Bottom* panels), we observe that the *In(2L)a* inversion region displays generally deeper coalescent times than the surrounding genomic background, with particular peaks in age near its breakpoints; consistent with prior observations of elevated diversity within this inversion (61). Previous age estimates of the inversion using phylogenomics have aged the origin of the inversion to more than two million years ago (62), before the origin of the *A. gambiae* complex of species. We find that the regions proximal to the inversion breakpoints show ages on the order of ∼1.1 million generations, consistent with this deep origin. This pattern likely reflects a combination of suppressed recombination between inverted and standard arrangements, as well as long-term balancing selection maintaining both arrangements in the population. While the deep origin of *In(2L)a* is well established, how such an ancient inversion has been maintained within populations, and whether its evolutionary history is uniform across its length, remains less well understood. The pronounced and spatially structured coalescent age peaks near the inversion breakpoints indicate that different regions of the inversion have experienced markedly different genealogical histories since its origin. This suggests that recombination suppression and long-term maintenance have acted heterogeneously across the inversion. More broadly, it demonstrates that population-scale genealogical inference can reveal biologically meaningful structure in the evolutionary dynamics of ancient chromosomal inversions beyond a single global age estimate.

Along chromosome 2L, a significant outlier is the well-studied *Rdl* locus, which confers resistance to dieldrin and other cyclodiene insecticides (63–65). Resistance to dieldrin appeared in Nigerian populations only ∼18 mo after the initial onset of insecticide control efforts in 1954 (66) as a single, dominant allele (67). Population genetic evidence points to a strong selective sweep at *Rdl* as well as a complex history of introgression that extends between species and inversion karyotypes (52). Our coalescent time estimates fill in this picture; we find clear evidence of recent selection at the locus, with short coalescent times among individuals in populations, such as Ghana, in which the resistant *Rdl* is at high frequency. Conversely in east African samples, where resistance is not found, coalescent times at the locus are indistinguishable from the local background.

Generally, our youngest time estimates at *Rdl* are on the order of hundreds to thousands of years, which would predate the introduction of broad insecticide use. Two things could be pushing back our dates here: i) it could be that the mutation was segregating at low frequency before the onset of selection, and ii) our estimates average over sites, and are not dating the origin of the *A296G* nonsynonymous mutation that confers resistance in *A. gambiae*.

Taken together, these applications show that cxt remains effective on large empirical datasets, including local genomic regions that deviate substantially from the evolutionary scenarios represented in its simulation-based pretraining, which acts as an implicit prior, such as targets of selective sweeps and long-term balancing selection. The Ag1000G analyses, in particular, highlight a practical advantage of simulation-based models: the ability to adapt to new evolutionary regimes with minimal targeted modification. By fine-tuning on high–$N_{e}$ simulations, reducing the window size to 200 bp, incorporating empirical missing-data masks, and adding a lightweight adapter for small sample sizes, cxt remained accurate in settings where traditional ARG MCMC and SMC-based methods face computational or modeling limitations. This flexibility, combined with amortized inference, positions cxt as a practical and scalable tool for population-genomics studies as community resources such as stdpopsim continue to expand to encompass additional species, demographic histories, and recombination landscapes.

In summary, we introduce a language-model-based inference engine designed to scale with the growing stdpopsim catalog. By directly learning a conditional stochastic process, cxt absorbs prior knowledge directly through simulation-based training, without requiring new analytic likelihood derivations for each scenario. As a result, cxt is a fast and accurate tool for TMRCA inference, and provides a flexible framework that can be adapted to novel evolutionary settings.

## Materials and Methods

### Next-Coalescence Prediction.

We introduce as our prediction task, *next-coalescence* prediction, which we define to mean the next point in time in which a focal (or pivot) pair of sequences find common ancestry as we move across (say from left to right) a sequence. Our method processes 500 genome windows (most commonly e.g. 500 × 2 Kb or 1Mb; or 500 × 200 bp or 0.1 Mb) of sequence. For the purposes of this paper we use a sample size of 25 diploid individuals, but this is arbitrary and we provide a method to train a light-weight adapter generalizing to required input sizes (or recommend subsampling and averaging for larger sample sizes). Our model focuses on a pivot pair of chromosomes, also known-as distinguished pairs in SMC++ terminology, from which we predict pairwise coalescence events, or more specifically a probability distribution from which the next coalescence event can be sampled for that pivot pair along the sequence for each of the 500 windows. This describes an iterative process by which the predicted coalescence time is concatenated with the mutational context, forming the next input to the model to predict the next coalescence event (Fig. 1). We refer to this procedure as *next-coalescence prediction*. We continue by describing the processing of the genotype matrix as obtained from coalescent simulation before presenting the architecture and listing the specific simulation parameterization next.

### Processing of Coalescent Simulations.

We begin with a genotype matrix $G\in Z^{2N\times M}$ containing 2N haploid sequences (from *N* diploid individuals) across *M* sites, obtained from coalescent simulations [here msprime (68)] or empirical data. All empirical analyses in this work use within-individual pivot pairs (the two haplotypes of one diploid, analogous to the PSMC setting), for which phasing is not required: Heterozygous and homozygous sites are determined by the genotype alone, so the XOR/XNOR features and SFS weights that constitute the model input are identical whether or not phase is known. Cross-individual pivot pairs, used here only in simulated benchmarks where phase is known by construction, would require phased haplotypes; in that setting, standard statistical phasing tools can be applied as a routine upstream step.

A high-level description is provided here, with an explicit version in *SI Appendix*, Algorithm 1 and a schematic overview in *SI Appendix*, Fig. S1. The core preprocessing consists of extracting mutation patterns for each pivot pair using logical operations—heterogeneous differences via XOR and homogeneous matches via XNOR—and then weights these pivot-pair vectors by their site-frequency spectrum (SFS) counts computed across all remaining samples. This converts the otherwise binary mutation indicators from the logical operations into coarse age-informed signals, ensuring that the inferred pairwise coalescence times are driven by the allele frequency spectrum present in the entire sample.

This procedure is applied in sliding windows of increasing size (4 to 128 Kb) with a 2 Kb stride, which allows for rescaling when inference is performed at different resolutions.

In summary, the model input consists solely of the genotype matrix and variant positions; from these, we derive windowed, SFS-weighted mutation patterns for each pivot pair, which the language model uses to estimate average coalescence times per window. These inputs arise naturally from msprime simulations or from empirical genotype data such as VCFs.

### Implementation of the Decoder-Only Architecture.

We implement a decoder-only transformer closely aligned with GPT-2 (69), rather than the original encoder–decoder formulation (70), and adapt it to the structure of population genetic data. In NLP, text is tokenized and mapped to embeddings via a learned lookup table; in contrast, the mutation patterns in genomic windows form a vast, effectively continuous space that makes discretization impractical. Instead of defining a bespoke tokenizer, we directly project SFS-weighted mutation patterns into the latent space using a fully connected embedding module (*SI Appendix*, Algorithm 2), which accepts tensors of shape B×Z×WS×K×N and outputs B×K×E. Here, *B* is batch size, *Z* the logical vector distinguishing heterozygous and homozygous states, *WS* the number of window sizes, *K* the number of windows, *N* the number of samples, and *E* the embedding dimension.

Coalescence times are represented by partitioning log-scaled values into 324 discrete intervals (*SI Appendix*, Table S1), each mapped through a small embedding table. The model then concatenates mutation and coalescence embeddings so that every context window carries both the local mutational signal and the discretized temporal state. This setup allows the same model to perform inference across diverse evolutionary scenarios without retraining, since the inputs already encode the relevant population-genetic context.

To encode genomic position, we adopt rotary positional embeddings (71), which efficiently capture spatial structure and require no adjustment under fixed-stride windows (e.g., 2 kb). The resulting sequence fed to the transformer consists of 500 mutation embeddings, a start token, and the coalescence embeddings, yielding a fixed latent length of 1,001; padding exists but is unused. A dropout rate of 0.1 is applied for regularization. Both narrow (single-scenario) and broad (multiscenario) models (cxt-narrow and cxt-broad respectively) share this architecture, with parameterization summarized in *SI Appendix*, Table S1. Further implementation details are available at https://github.com/kr-colab/cxt.

### Sample-Size and Accessibility Adaptation.

cxt models are trained at a fixed sample size, which determines the number of SFS channels used to represent the mutational context. Empirical datasets may contain fewer samples than the pretrained model (e.g. the Ghana population in Ag1000G), and retraining the full model for each sample size would be inefficient. To enable transfer to smaller cohorts, we introduce a lightweight input-adaptation stage that operates before the pretrained decoder-only transformer.

#### Sample-size adapter.

The adapter maps SFS features computed from a reduced cohort to the dimensionality expected by the pretrained model. After this transformation, data are processed by the original projection layers and transformer without further modification. In this way, the adapter modifies only how mutational information is presented to the model, while preserving the pretrained representation of the coalescent process.

The adapter is implemented as a small residual neural network applied independently to each genomic window. It consists of layer normalization followed by a gated bottleneck that rescales and recombines SFS channels derived from the smaller cohort. A residual connection projects the input directly to the target dimensionality, and a learnable scaling parameter initialized near zero controls the magnitude of the adapter’s contribution. As a result, the adapter behaves close to an identity mapping at the start of training and fine-tunes in a stable manner.

#### Training procedure.

Adapter training is performed by freezing all parameters of the pretrained decoder-only transformer and updating only the adapter parameters. The training objective is identical to that of the full model, namely prediction of the local pairwise coalescence time in each genomic window. Because the adapter contains far fewer parameters than the backbone, it converges rapidly and can be trained in a small number of epochs using simulations generated at the target sample size.

To accommodate small but systematic shifts in input statistics, we additionally unfreeze the LayerNorm parameters and the final transformer blocks and train them at a substantially reduced learning rate. This restricted fine-tuning improves stability when transferring to smaller cohorts while avoiding degradation of the pretrained coalescent prior.

#### Fine-tuning to genomic inaccessibility.

Empirical genomes contain extended regions that are systematically uncallable, producing structured missingness patterns that differ from idealized simulations. To account for this, we fine-tune the adapted model on training examples in which genomic inaccessibility is imposed explicitly using an empirical accessibility mask.

For each simulated tree sequence, we select a contiguous genomic segment and delete intervals corresponding to inaccessible sites from the tree sequence, ensuring that both mutational and genealogical information reflect the empirical observation process. In parallel, we compute the fraction of inaccessible bases per window across multiple scales and inject this signal into a dedicated input channel, providing the model with explicit information about local callability. This fine-tuning step uses the same training objective as above and updates the same restricted set of parameters (adapter, LayerNorms, and final transformer blocks), allowing the model to retain its pretrained coalescent representation while calibrating to irregular observation patterns in real genomes.

### Calibration of the Molecular Clock.

Although cxt is mutation-rate agnostic during training, its raw TMRCA predictions must be placed on an absolute timescale for demographic interpretation. We therefore apply a simple calibration step that rescales predicted TMRCAs so that the expected nucleotide diversity matches the observed diversity across the full context window. This correction is typically negligible when the data resemble the training scenarios, but becomes useful under atypical mutation rates or demographic histories. To avoid collapsing variability across replicates, we treat the correction factor probabilistically rather than deterministically. A full derivation, including the Poisson–Gamma model used to sample per-replicate correction factors, is provided in *SI Appendix*, Figs. S2 and S3.

### Estimating Population Size from Coalescence Rates.

To convert predicted TMRCAs into population size trajectories, we estimate the instantaneous coalescence rate on a logarithmic time grid and invert it to obtain $N_{e}\left(t\right)=1/\left(2\lambda \left(t\right)\right)$. This provides a nonparametric demographic reconstruction directly from the model’s TMRCA samples. Details, including windowing, discretization, and treatment of the tail interval, appear in *SI Appendix*.

### Datasets and Training Description.

Full details of the training/validation parameter sets (narrow versus broad; stdpopsim v0.2 for training and v0.3 reserved for validation), along with exact commands, are provided in *SI Appendix* and in the online manual. Models were implemented in PyTorch Lightning and trained with AdamW (base learning rate $3\times 10^{-4}$) with cosine annealing; datasets were stored in float16 for efficiency. Fine-tuned variants were trained for two epochs with a ten-fold reduced learning rate. Inputs follow a fixed-length mutation context paired with coalescence tokens, trained with a causal NLL objective. We use a translation-aware fused-causal mask so coalescence predictions attend to all prior mutations and past coalescences, while mutation predictions attend to all prior tokens.

### Environmental Considerations.

Although GPUs typically draw more instantaneous power than CPUs (e.g., ∼250 to 400 W for an NVIDIA A100 versus ∼225 W for a high-end server CPU), they substantially reduce wall-clock time for the parallel inference workloads used by cxt (training requires on the order of 10 h on three A100s). Because amortized inference is insensitive to recombination-rate variation, GPU acceleration can reduce total energy use and $CO_{2}$ emissions relative to CPU-based likelihood methods, particularly in large-$N_{e}$ regimes.

## Supplementary Material

Appendix 01 (PDF)

## Data Availability

(50)Simulation code, model training workflows, and accompanying documentation are available under a CC-BY-NC 4.0 license at https://github.com/kr-colab/cxt (72), with additional resources at https://cxt.readthedocs.io/en/latest/ (73). Publicly available data from the 1000 Genomes Project were used for evaluation.
