# Supplementary material for: Coalescence and translation: A language model for population genetics
Source: Proc Natl Acad Sci U S A. 2026 Apr 10;123(15):e2518956123. doi: 10.1073/pnas.2518956123 (PMC13079918; doi:10.1073/pnas.2518956123)
Supplement: Supplementary file 1 — Appendix 01 (PDF) [file pnas.2518956123.sapp.pdf]

Supporting Information for

Coalescence and Translation: A Language Model for Population Genetics

Kevin Korfmann, Nathaniel S. Pope, Melinda Meleghy, Aurélien Tellier, and Andrew D. Kern

Kevin Korfmann.
E-mail: korfmann@sas.upenn.edu

This PDF file includes:

Supporting text
Figs. S1 to S15
Tables S1 to S6

Supporting Information Text

Supplementary Methods

Things We Tried.

Preprocessing mutational patterns We explored representing local genealogical configurations via a discrete "codebook" of mutation patterns, allowing the model to operate on higher-level coalescent motifs rather than raw mutation arrays. In practice, the combinatorial growth with sample size and window length made codebook learning brittle, non-extensible, and poorly suited for scenarios requiring inference of multiple coalescent times. Performance degraded sharply outside the specific training regimes.

Hand-rolled convolutions via multi-scale windows. To mimic convolutional behavior, the current model uses multiple window sizes in the embedding stack. Single-scale tokenization consistently undercaptured rare, localized events such as sharp TMRCA dips. Although we considered adding true convolution operators, the multi-scale approach remained the simplest, at the cost of increased storage and additional input processing requirements.

Architectural upgrades with limited impact. We evaluated more advanced attention variants (linear attention, multi-query attention) but observed negligible gains given our short contexts (about 500 tokens per 1 Mb). These optimizations mainly benefit very long-sequence models, and the added engineering complexity was not justified.

Splitting the encoder and decoder. We also considered a cleaner encoder-decoder separation for improved maintainability and the possibility of halving context length by compressing the mutation stream prior to temporal decoding. While promising—especially in light of simpler key-value caching—the monolithic GPT-like architecture appeared conceptually simpler, though we may return to this design in future models.

Training mixtures without repetition. Early training experiments drew demographies randomly without repetition across epochs. This prevented the model from seeing the same statistical structure often enough to form stable internal representations and led to degraded inference accuracy. Reintroducing repetition—training many iterations of the same demography with different random seeds—proved essential for stable learning and effective generalization.

Algorithmic Details of Preprocessing. For processing the input the complete algorithmic details are provided in Algorithm 1 below. This describes the internal construction of the tensor to be ingested by our model.

Algorithm 1 ProcessGenotypeMatrixAndPositions (Vectorized)

Require: $G \in \mathbb{Z}^{N \times M}$ ▷ Genotype matrix (N samples, M sites)
Require: $P \in \mathbb{R}^{M}$ ▷ Site positions ($P[j]$ is the position of site j)
Require: L ▷ Sequence length (total genomic range)
Require: $W_m = [2, 8, 32, 64]$ ▷ Window multipliers
Require: $w = 4000$ ▷ Base window size
Require: $s = 2000$ ▷ Step size
Require: A, B ▷ Pivot sample indices
Require: `xor_ops` ▷ Logical operation: XOR or XNOR
Ensure: $X \in \mathbb{Z}^{2 \times |W_m| \times K \times N}$ ▷ Feature matrix, with $K = \lceil L/s \rceil$

// **Step 1: Compute Logical Frequencies (Vectorized)**
$F \leftarrow \text{sum}(G, \text{axis} = 0)$ ▷ Vectorized sum over samples; $F \in \mathbb{Z}^{1 \times M}$
$L\_val_{XOR} \leftarrow G[A, :] \oplus G[B, :]$
$L\_val_{XNOR} \leftarrow \neg\big(G[A, :] \oplus G[B, :]\big)$
$F\_logic[0, :] \leftarrow F \odot L\_val_{XOR}$ ▷ For XOR
$F\_logic[1, :] \leftarrow F \odot L\_val_{XNOR}$ ▷ For XNOR
// **Step 2: Define Sliding Windows (Vectorized Boundaries)**
$K \leftarrow \lceil L/s \rceil$
$\mathbf{start} \leftarrow s \cdot [\,0, 1, \ldots, K-1\,]$ ▷ Vector of window start positions
for $i \leftarrow 1$ **to** $|W_m|$ **do**
    $W_{\text{end}}[i] \leftarrow w \cdot W_m[i]$
$\mathbf{end} \leftarrow \min\Big(\mathbf{start} + W_{\text{end}},\ L\Big)$ ▷ Broadcasted over multipliers
For each window index k and multiplier index i, define

$$W[k, i] = \{\, j \mid P[j] \in [\,\mathbf{start}[k],\ \mathbf{end}[i]\,]\,\}$$

// **Step 3: Compute Site Frequency Spectrum (Vectorized Bincount)**
for $d \in \{0, 1\}$ **do**
    for $i \leftarrow 1$ **to** $|W_m|$ **do**
        for $k \leftarrow 0$ **to** $K - 1$ **do**
            $X[d, i, k, :] \leftarrow \text{bincount}\Big(F\_logic[d, W[k, i]],\ \text{minlength} = N\Big)$
return X

The input module projecting SFS values per window of specified size into latent space in parallel for 500 windows, 4 window sizes, and 2 states (heterogeneous vs. homogeneous) and B batches.

Algorithm 2 ProjectionReplacingEmbeddingTable

Require: $x \in \mathbb{R}^{B \times Z \times WS \times K \times N}$, parameters W_1, b_1, W_2, b_2, weights w_1, w_2
Ensure: $\hat{x} \in \mathbb{R}^{B \times K \times d}$
$x[\ldots, 0] \leftarrow 0$
$\phi_1(x) \leftarrow \text{GELU}(W_1\, x + b_1)$
$\phi_2(x) \leftarrow W_2\, x + b_2$
$\hat{x} \leftarrow w_1\, \phi_1(x) + w_2\, \phi_2(x)$
$\hat{x} \leftarrow \text{reshape}(\hat{x}, [B,\ K,\ E])$
return $\hat{x}$

Model Configuration. Hyperparameter specifications of our narrow and broad models.

Datasets and Training Details. The following sections provide detailed information on dataset construction, including coalescence parameter initialization, dataset composition, and training procedures. Training parameters are organized into distinct sets: a single parameterization for the narrow model, designed to approximate a constant-size human demographic scenario, and an expanded set for the broad model encompassing most demographic models from `stdpopsim` v0.2. Models from `stdpopsim` v0.3 are reserved exclusively for validation and were not used during training.

We implemented our models using PyTorch Lightning to streamline code management, including multi-GPU training and loss tracking. Matrix multiplication precision was set to float32 (medium), relying on PyTorch Lightning's internal precision handling, and we did not use additional manual autocasting with 'bf16-mixed' during training. To optimize memory efficiency and storage, datasets were stored in float16 format.

The model was trained for five epochs using the `AdamW` optimizer with a learning rate of 3×10^{-4}, which was gradually decreased during training via cosine annealing.

Table S1. Token-Free Decoder Model Configuration

Parameter	Narrow Model	Broad Model
Number of Layers (n_{layer})	6	10
Number of Attention Heads (n_{head})	4	4
Embedding Dimension (n_{embd})	400	400
Dropout Rate	0.1	0.1
Bias in Linear Layers	False	False
Number of Samples per Forward Pass	50	50
Sample Scale Embedding Factor	2	2
Output Dimension ($\text{output}_{\text{dim}}$)	326	326
Combined Feature Dimension ($\text{combined}_{\text{dim}}$)	1001	1001
**Total parameters** in millions	≈ 11.7	≈ 19.5

Fine-tuned models were trained for only two epochs on their respective datasets, using a learning rate reduced by a factor of ten. For more information about specific training commands, we refer to the online manual.

Each training sample of a batch consists of 500 windows, followed by a start token, which is the second row of the embedding table for time discretization (the first row is reserved for potential padding, but is not used in this implementation). This is then followed by 500 coalescent times. The target sequence is simply a right-shifted version of the coalescent times, beginning with the first coalescent event. Accordingly, the model is trained in the same manner as standard language models, using negative log-likelihood loss and a causal mask. The causal mask has been adapted for translation within the relevant attention layers. Specifically, we used a fused-causal attention mask, which lets the decoder distinguish mutation from coalescence tokens when "looking back" over the sequence: when predicting a mutation, the model can attend to all previous tokens, but when predicting a coalescence, it can attend to all earlier mutations and only to past (not future) coalescences. This preserves strict causal ordering for sequence decoding while keeping the full, fixed mutation history visible at every step.

We organized our training data into four distinct sets. The first is a large base dataset, used to train the narrow model, with parameters chosen to approximate human simulations. This set consists of a single demographic scenario with constant population size (see Table S2). We also performed fine-tuning of the narrow model for a few auxiliary models (Table S3). The third dataset is the `stdpopsim` v0.2 dataset (see Table S4), the main dataset used in this study that we call the broad model. The final dataset comprises `stdpopsim` v0.3 models, which are used for evaluation (see Table S5).

Calibration of the Molecular Clock. `cxt` output is run in replicates (default 15), which together provide a distribution over TMRCA trajectories. Although the language model is agnostic to mutation rate (in the sense that this is not provided as an explicit input, and the model sees data generated under many mutation rates during training), it will generally be desirable to condition on a particular mutation rate/molecular clock for N_e calibration purposes. This is done by scaling the raw predictions such that expected nucleotide diversity matches the observed nucleotide diversity across the full context (e.g. the 1Mb sequence). Empirically, this first-order correction is most helpful when the model is applied to data that are atypical relative to the training simulations (see Figure S2). Crucially, the relative TMRCA is highly accurate even in out-of-distribution scenarios, so that the first order correction is generally sufficient to make bias and mean squared error constant across a wide range of scenarios.

One issue is that a deterministic first-order correction will reduce variability in `cxt`'s "posterior" samples—for example, TMRCA trajectories that are constant across the windows but have distinct values across replicates, would be all collapsed to the same value. Thus, we model the correction factor probabilistically to preserve replicate variability. For a given pivot pair i, we assume the observed mutation count y_i follows a Poisson model:

$$y_i \sim \text{Pois}\left(2\mu c_i s \sum_j t_{ij}\right),$$

where s is the window size, μ is the mutation rate, t_{ij} is the predicted TMRCA in window j of replicate i, and c_i is the

Table S2. Base Dataset (Approx. for Human Parameters)

Parameter	Value
Population Model	Constant Demography
Population Size	2×10^4
Mutation Rate	1.29×10^{-8} per Base
Recombination Rate	1.28×10^{-8} per Base
Simulations	2×10^6
Sequence Length	1 Mb

Table S3. Base Dataset Extension (auxiliary Dataset): Model Parameters *

Model	Abbreviation	Values	Description
`ne.constant`	N_e	$10^4,\ 2\times10^4,\ 4\times10^4$	Effective population size
	μ	$1\times10^{-8}, 5\times10^{-8}$	Mutation rate per base
	r	$1\times10^{-8}, 5\times10^{-8}$	Recombination rate per base
`ne.sawtooth`	N_e	$10^4,\ 2\times10^4,\ 4\times10^4$	Effective population size
	Magnitude	`strong, medium, weak`	Amplitude of N_e oscillations
	μ	$1\times10^{-8}, 5\times10^{-8}$	Mutation rate per base
	r	$1\times10^{-8}, 5\times10^{-8}$	Recombination rate per base
`island.3pop`	N_e	$10^4,\ 2\times10^4,\ 4\times10^4$	Effective population size
	m	$0.05,\ 0.2$	Migration rate between islands
	μ	1×10^{-8}	Mutation rate per base
	r	1×10^{-8}	Recombination rate per base
`hard_sweeps`	N_e	$10^4,\ 2\times10^4,\ 4\times10^4$	Effective population size
	p_s	$2.5\times10^{-5},\ 1\times10^{-5},\ 7.5\times10^{-5}$	Selected site position or selection coefficient
	μ	$1\times10^{-8}, 5\times10^{-8}$	Mutation rate per base
	r	$1\times10^{-8}, 5\times10^{-8}$	Recombination rate per base

* Within each model, all-vs-all parameter combinations were simulated; the only exception (when $\mu = 5\times10^{-8}$ & $r = 5\times10^{-8}$) was skipped due to long simulation times.

per-replicate correction factor. Under an improper constant prior, this yields a posterior:

$$c_i \sim \text{Gamma}\left(y_i + 1,\ 2\mu s \sum_j t_{ij}\right).$$

We then sample an independent c_i for each replicate and apply it to all windows in that replicate. This propagates uncertainty from diversity estimation into the corrected predictions. In practice, the difference is negligible when mutation counts are large, as the posterior concentrates around the maximum likelihood estimate. However, when mutation counts are sparse or when the model produces flat TMRCA profiles, this stochastic correction helps avoid degeneracies (e.g., where there are no mutations and the MLE is undefined).

Calibration of Approximate Posteriors. While the results above focus on point accuracy, `cxt` also samples TMRCA trajectories from an approximate posterior, enabling uncertainty quantification in addition to producing point estimates (e.g. the posterior mean). We therefore tested empirically whether the marginal approximate posteriors are (i) well-calibrated in the sense of providing correct frequentist coverage of the true TMRCA, and (ii) consistent, in the sense that the posterior concentrates as the amount of mutational information increases.

To assess these properties, we first simulated 1,000 completely independent pivot pairs under three demographic scenarios, including one drawn from the out-of-training `stdpopsim` v0.3 release (*Oryza sativa*). For each pair, we computed posterior intervals for the TMRCA in each 2 kb window using 100 `cxt`-sampled trajectories. For an exact posterior, an interval containing a fraction α of posterior mass contains the true value with probability α. We therefore evaluate calibration by computing, across windows, the empirical fraction of true TMRCAs that fall within posterior intervals of varying nominal mass. Second, we evaluate consistency by measuring how the average posterior variance changes as a function of mutation rate, scaled by 0.5, 1, and 2 relative to the species-specific mutation rate reported in the `stdpopsim` catalog. Both of these measures are calculated with respect to the position within the full 1Mb prediction window, since we expect performance to degrade near the boundaries where the local mutational context is truncated.

Across three distinct parameter regimes, we find that `cxt` produces generally well-calibrated posterior samples across most positions within the 1 Mb prediction window (Supplementary Figure S9, top row). These approximate posteriors are typically slightly over-concentrated at high nominal coverage (e.g., intervals containing 95% of the posterior mass contain the true TMRCA roughly 92% of the time), but are otherwise remarkably stationary: the empirical coverage declines appreciably only in the final 2 kb window. This drop appears to be driven by a tendency of the trained model to predict occasional jumps in the last window, which can be addressed in practice by omitting that window prior to centering the predictions. Consistent with posterior concentration, increasing (decreasing) mutational density, causes the posterior intervals to contract (expand) reflecting increased (decreased) information (Supplementary Figure S9, bottom row). As expected, windows near the boundaries also exhibit greater uncertainty, consistent with truncation of the local mutational context.

Table S4. `stdpopsim` **dataset (v0.2): Simulations configuration for training**

Species	Demographic Model	Genetic Map	Simulations
Aedes aegypti	PieceWiseConstant		6.0×10^4
Anas platyrhynchos	MallardBlackDuck_2L19		5.0×10^3
Anolis carolinensis	PieceWiseConstant		1.0×10^3
Anopheles gambiae	GabonAg1000G_1A17		2.0×10^4
Arabidopsis thaliana	SouthMiddleAtlas_1D17	SalomeAveraged_TAIR10	1.0×10^5
Arabidopsis thaliana	African2Epoch_1H18	SalomeAveraged_TAIR10	1.0×10^5
Arabidopsis thaliana	African3Epoch_1H18	SalomeAveraged_TAIR10	1.0×10^5
Bos taurus	HolsteinFriesian_1M13		2.0×10^5
Caenorhabditis elegans	PieceWiseConstant	RockmanRIAIL_ce11	2.0×10^5
Canis familiaris	PieceWiseConstant	Campbell2016_CanFam3_1	2.0×10^5
Drosophila melanogaster	African3Epoch_1S16	ComeronCrossoverV2_dm6	1.0×10^3
Drosophila melanogaster	OoA_2L06	ComeronCrossoverV2_dm6	1.0×10^3
Drosophila sechellia	PieceWiseConstant		6.0×10^4
Gasterosteus aculeatus	PieceWiseConstant		2.0×10^5
Helianthus annuus	PieceWiseConstant		6.0×10^4
Homo sapiens	OoAExtNeaAdmixturePulse_3I21	HapMapII_GRCh38	2.0×10^5
Homo sapiens	OoA_3G09	HapMapII_GRCh38	2.0×10^5
Homo sapiens	OoA_2T12	HapMapII_GRCh38	2.0×10^5
Homo sapiens	Africa_1T12	HapMapII_GRCh38	2.0×10^5
Homo sapiens	AmericanAdmixture_4B18	HapMapII_GRCh38	2.0×10^5
Homo sapiens	OoAArchaicAdmixture_5R19	HapMapII_GRCh38	2.0×10^5
Homo sapiens	Zigzag_1S14	HapMapII_GRCh38	2.0×10^5
Homo sapiens	AshkSub_7G19	HapMapII_GRCh38	2.0×10^5
Homo sapiens	OoA_4J17	HapMapII_GRCh38	2.0×10^5
Homo sapiens	Africa_1B08	HapMapII_GRCh38	2.0×10^5
Pan troglodytes	BonoboGhost_4K19		2.0×10^5
Papio anubis	SinglePopSMCpp_1W22	Pyrho_PAnubis1_0	2.0×10^5
Pongo abelii	TwoSpecies_2L11	NaterPP_PonAbe3	2.0×10^5

Table S5. `stdpopsim` **dataset (v0.3): Simulations configuration for evaluation**

Species	Demographic Model
Gorilla gorilla	GorillaGhost_5P23
Oryza sativa	BottleneckMigration_3C07
Rattus norvegicus	PieceWiseConstant
Sus scrofa	PieceWiseConstant

Together, these findings indicate that the approximate posteriors produced by `cxt` provide reliable uncertainty quantification for predicted coalescence times, across demographic scenarios and mutational densities. This is a key strength of the method, particularly because posterior sampling is so computationally efficient.

Estimating Population Size via Instantaneous Inverse Coalescence Rates. We estimate the instantaneous coalescence rate from a set of ancestor coalescence times by discretizing time into logarithmically spaced windows ($T_{\max} = 40$). Specifically, we define the time grid using

$$\texttt{time\_windows} = \text{log-space}\left(2, \lfloor \log_{10}(T_{\max}) \rfloor, N+1\right)$$

where $T_{\max}$ is the maximum simulation time and N is the number of desired windows. We then explicitly set the first window edge to 0 by assigning `time_windows[0] = 0.0`. This creates N non-overlapping intervals $[t_0, t_1), [t_1, t_2), \ldots, [t_{N-1}, t_N)$

that cover the relevant timescale on a logarithmic scale, providing finer resolution for more recent coalescent events.

Let $T_1, T_2, \ldots, T_n$ be the observed coalescence times. For each window $[t_i, t_{i+1})$, we compute the proportion of events falling into that bin, giving a discretized probability density function (PDF), denoted p_i. The corresponding cumulative distribution function (CDF) is $F(t_i) = \sum_{j<i} p_j$. Assuming a memoryless coalescence process (i.e., a Poisson process), the instantaneous rate $\lambda(t)$ satisfies:

$$\lambda(t) = \frac{f(t)}{1 - F(t)}$$

where $f(t)$ is the density and $1 - F(t)$ is the survival function. Integrating this rate over the interval $[t_i, t_{i+1})$ gives:

$$\int_{t_i}^{t_{i+1}} \lambda(t)\, dt = \log\left(\frac{1 - F(t_i)}{1 - F(t_{i+1})}\right)$$

and dividing by the window width $\Delta t = t_{i+1} - t_i$, we estimate the average coalescence rate in the interval as:

$$\hat{\lambda}_i = \frac{1}{\Delta t} \log\left(\frac{1 - F(t_i)}{1 - F(t_{i+1})}\right)$$

For the final window (where the survival function becomes too small), we estimate the rate via the inverse of the mean residual coalescence time:

$$\hat{\lambda}_{\text{last}} = \left(\mathbb{E}[T - t_{\text{last}} \mid T \geq t_{\text{last}}]\right)^{-1}$$

This approach provides a nonparametric, smoothed estimate of the coalescence rate as a function of time, suitable for demographic inference.

Computational Efficiency and Scaling. We compared wall-clock runtime for `cxt`, SMC++, and `Singer` across increasing numbers of inferred pairwise coalescence trajectories (Figure S6). Because these methods differ fundamentally in how inference is performed, runtime is evaluated separately from statistical accuracy. `SMC++` achieves fast local decoding, but total runtime is dominated by preprocessing and composite-likelihood optimization, both of which increase with dataset size. Note that `SMC++` also assumes unphased samples—a constraint that could be addressed by restructuring the VCF file itself, but which we omit here, as runtimes are already large. In contrast, `cxt` performs fully amortized inference: after training, runtime scales approximately linearly with the number of inferred pairs and shows near-linear speedups with additional GPUs. Runtime is largely insensitive to recombination rate, enabling predictable performance across parameter regimes, but preprocessing overhead is noticeable when going beyond 50 samples. `Singer` shows competitive accuracy but substantially higher runtime, with sensitivity to recombination parameters reflecting its reliance on explicit genealogical sampling. Overall, these results highlight a qualitative difference in computational scaling: while likelihood-based and MCMC-based methods incur increasing cost as genealogical complexity grows, `cxt` shifts this cost to training, enabling fast inference at application time, while as of now being constrained by the pairwise binomial scaling with the number of samples.

Table S6. Mean squared error (MSE; lower is better) for three approaches across two demographic scenarios. We report results separately for the narrow and broad variants of `cxt` (that `cxt`-adapter uses the broad model as its basis) alongside Singer+Polegon and `SMC++` reflecting training on a single demographic regime (constant size) versus a fluctuating demographic history (sawtooth), respectively.

Method	Constant	Sawtooth
`cxt`-narrow	0.2531	0.7496
`cxt`-broad	–	**0.1796**
`cxt`-adapter	0.2631	0.1946
Singer+Polegon	**0.2470**	0.2129
`SMC++`	0.8685	1.7919

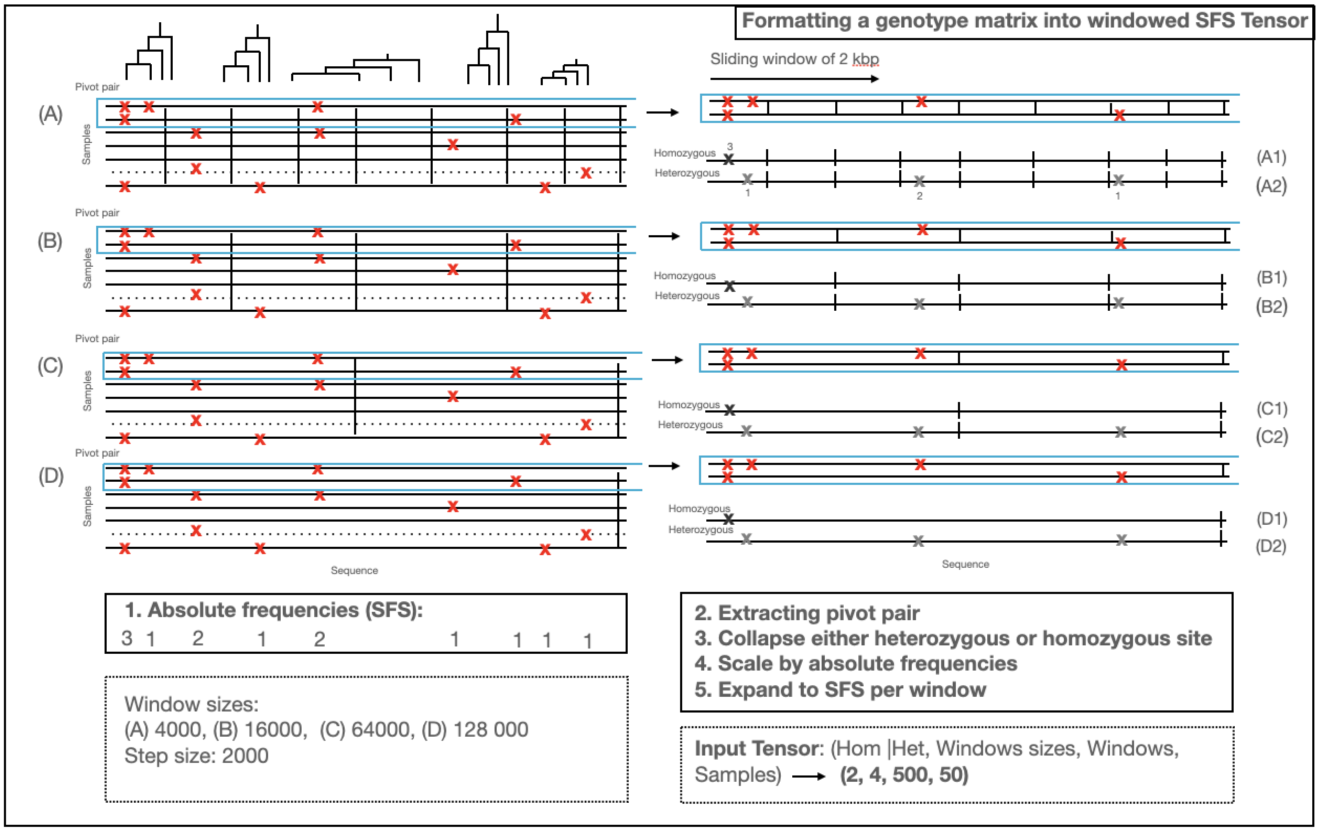

Fig. S1. Processing of a genotype matrix, as detailed in Algorithm 1. This figure provides a schematic overview. Starting at (**A**), we compute absolute variant frequencies across 50 samples (a fixed hyperparameter) (1). A genomic region is divided into 500 windows. We focus on a pivot pair (framed in blue), extract it, and classify sites as heterogeneous or homogeneous. Each site is scaled by its SFS class, and each window is expanded into the full sparse SFS dimensions of 50 (see input tensor, bottom right). This process repeats for three additional windows (**B–D**), yielding a complete input tensor of shape (2, 4, 500, 50) per pair, with the SFS aggregating information across all samples.

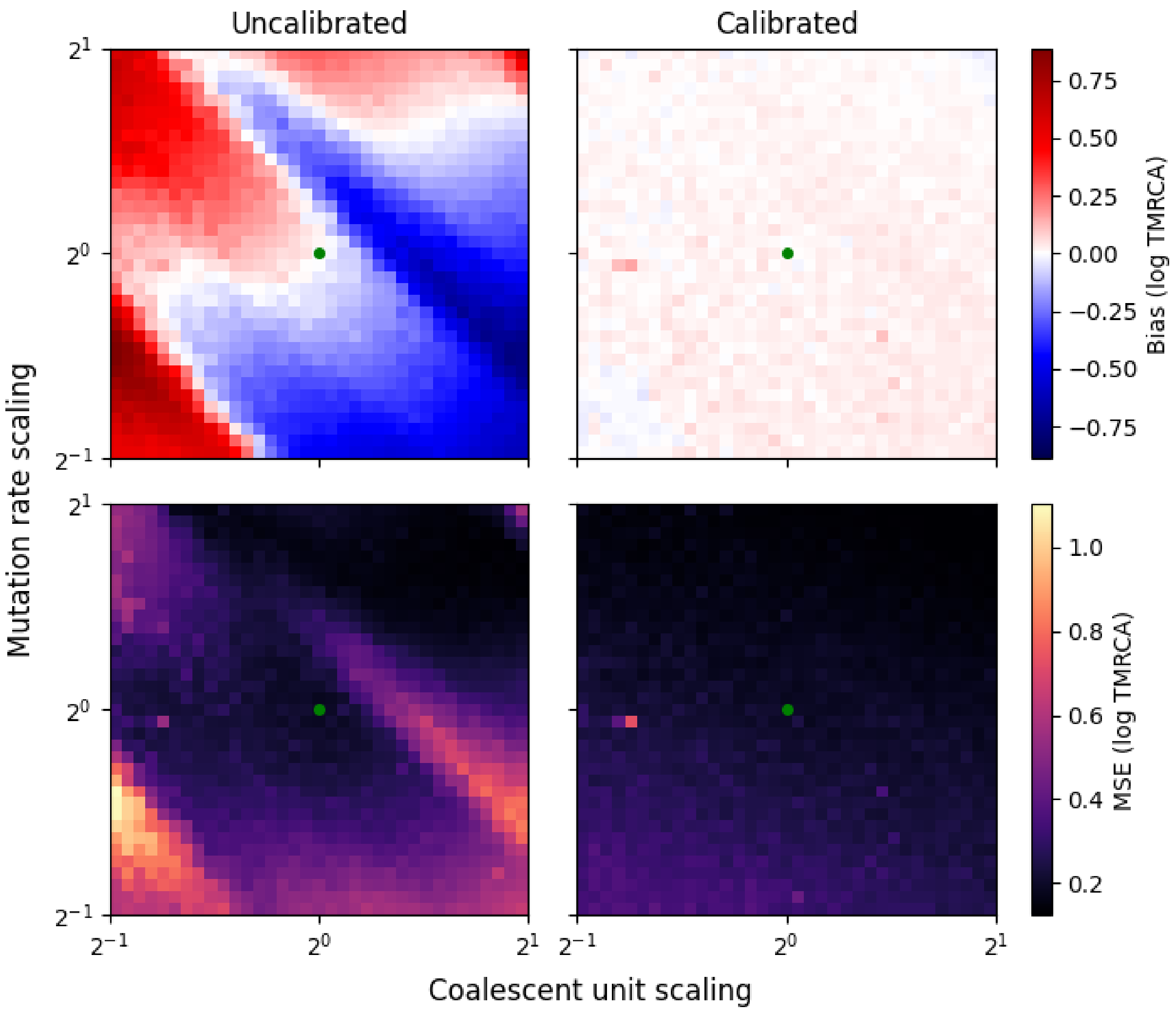

Fig. S2. The impact of calibrating the molecular clock using observed nucleotide diversity (see section "Calibration of Molecular Clock" in methods), using simulations from perturbations of the Zigzag model in the `stdpopsim` catalog. The x-axis is a scaling of the Markov generator for the coalescent process, that increases the average TMRCA while keeping the shape of the site frequency spectra constant. The y-axis is a scaling of the mutation rate. The two columns correspond to uncalibrated and calibrated `cxt` predictions (e.g. in the former, the language model is predicting absolute TMRCA; and in the latter relative TMRCA). The two rows are bias (top) and mean squared error (bottom) relative to the true TMRCA, calculated from 30 independent simulations per grid cell. The unperturbed demography (which the language model sees during training) is the green point in the center of each heatmap. As the model is perturbed, the uncalibrated `cxt` predictions become substantially biased in a fashion that is nonlinear with regards to the magnitude of the perturbations (left column); reflecting the fact that the TMRCAs, mutation rate, and recombination rate are not jointly identifiable. However, relative changes in TMRCA are predicted accurately, so that centering the TMRCA trajectories around the observed nucleotide diversity for each pivot pair (e.g. conditioning on the mutation rate) removes the bias regardless of the degree of perturbation. Thus, after calibration (right column) the MSE for `cxt` predictions follows a gradient of mutational density.

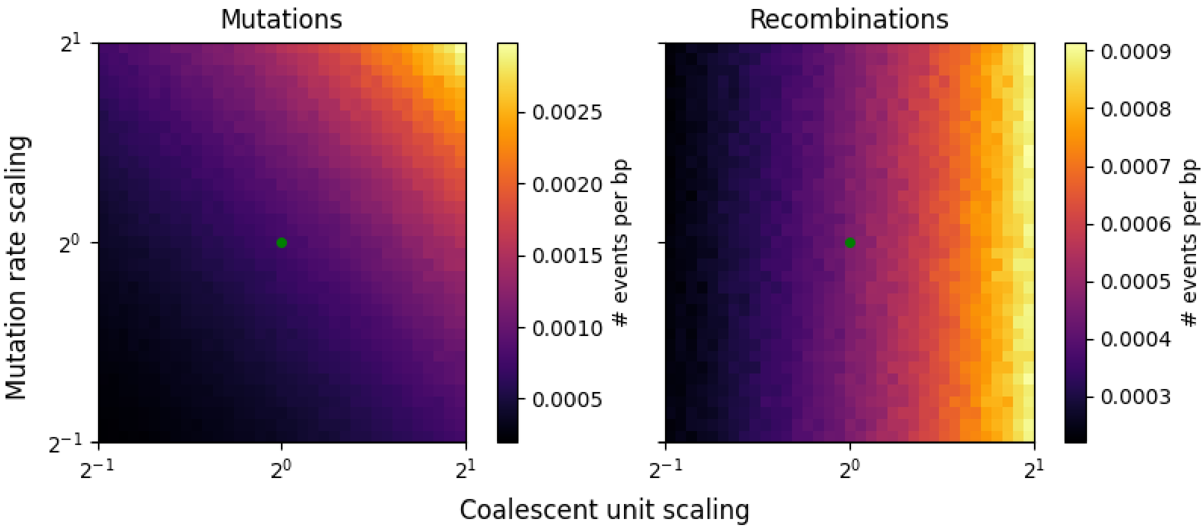

Fig. S3. The density of mutations and recombination events across perturbations of the Zigzag `stdpopsim` model, for the simulations shown in Figure S2.

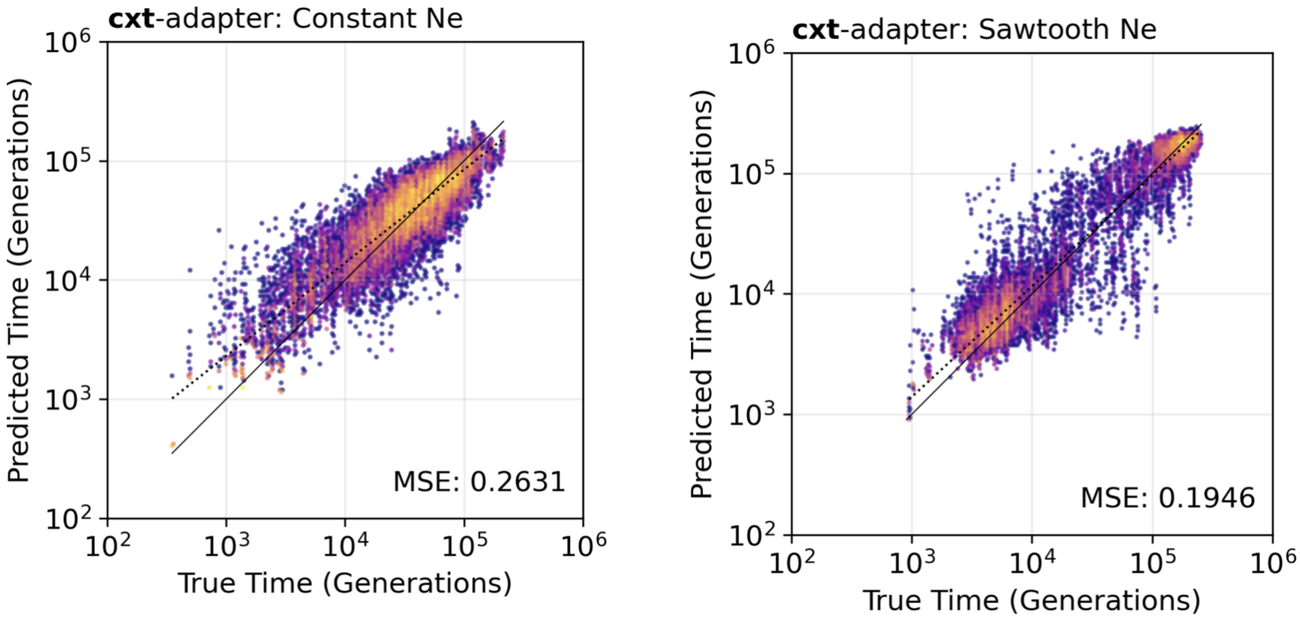

Fig. S4. `cxt`'s broad model fine-tuned with a low-parameter adapter to account for small sample sizes. On the left, `cxt` is demonstrated on a constant demography, while on the right the sawtooth scenario is presented.

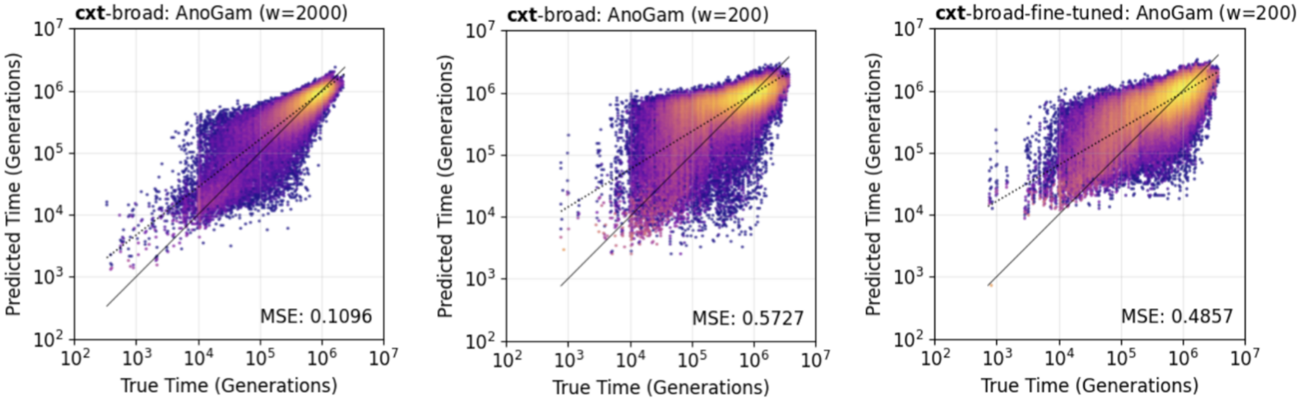

Fig. S5. `cxt`'s broad model (left) on `stdpopsim` AnoGam parameterization at window resolution 2 Kb, in the middle at 0.2 Kb and after fine-tuning on large `stdpopsim` species larger than 100 k N_e.

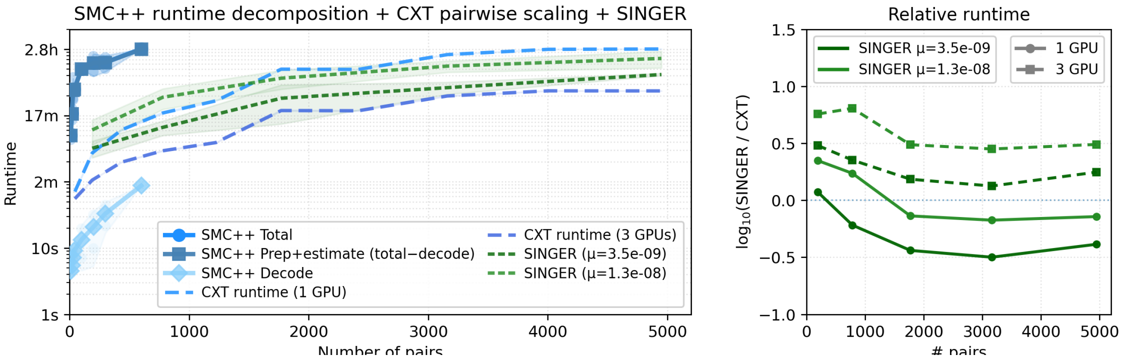

Fig. S6. Wall-clock runtime (log scale) is shown as a function of the number of inferred haplotype pairs for SMC++ (solid; decomposed into total, preprocessing+estimate, and decoding posterior), `cxt` (dashed blue; 1 to 3 GPUs), and `Singer` (dashed green; two mutation-rate settings). Right panel shows the fold difference between `Singer` and `cxt` with respect to recombination rate or number of GPUs.

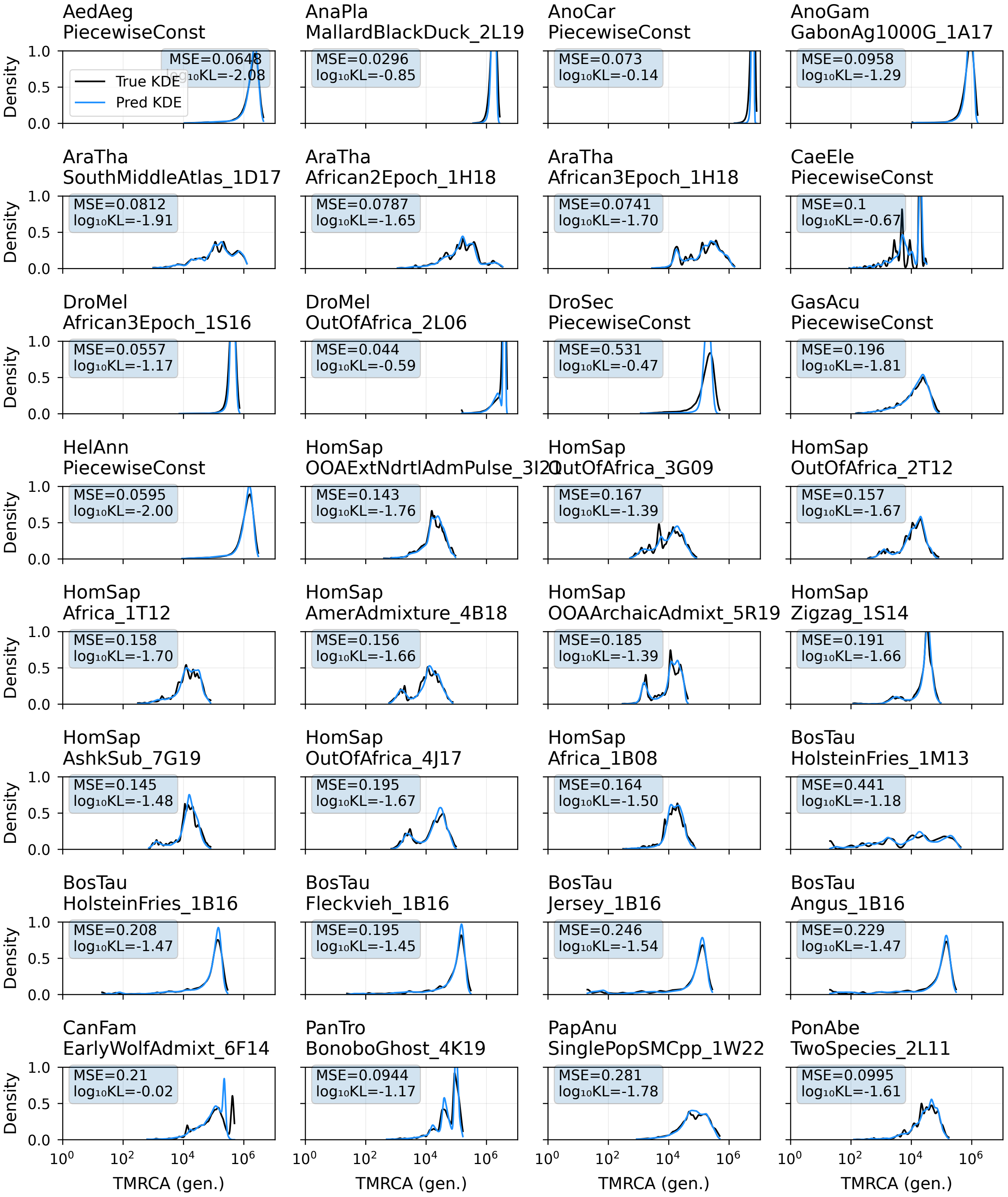

Fig. S7. Evaluation of marginal coalescence distribution inference (blue line) against the true distribution (black line), probing the model's capacity to distinguish among many scenarios based on context alone. The broad model's ability to infer a `stdpopsim` v0.2 coalescence distribution is tested, which includes diverse mutation and recombination rates, as well as demographic scenarios. This distribution is obtained by aggregating pairwise inferences and visualizing the result as a kernel density for each scenario. All results shown are from new simulations, not included in the original training dataset. No genetic map was used for the inference shown here; the model itself was trained with and without genetic maps. For inferences with an underlying genetic map see Figure S8.

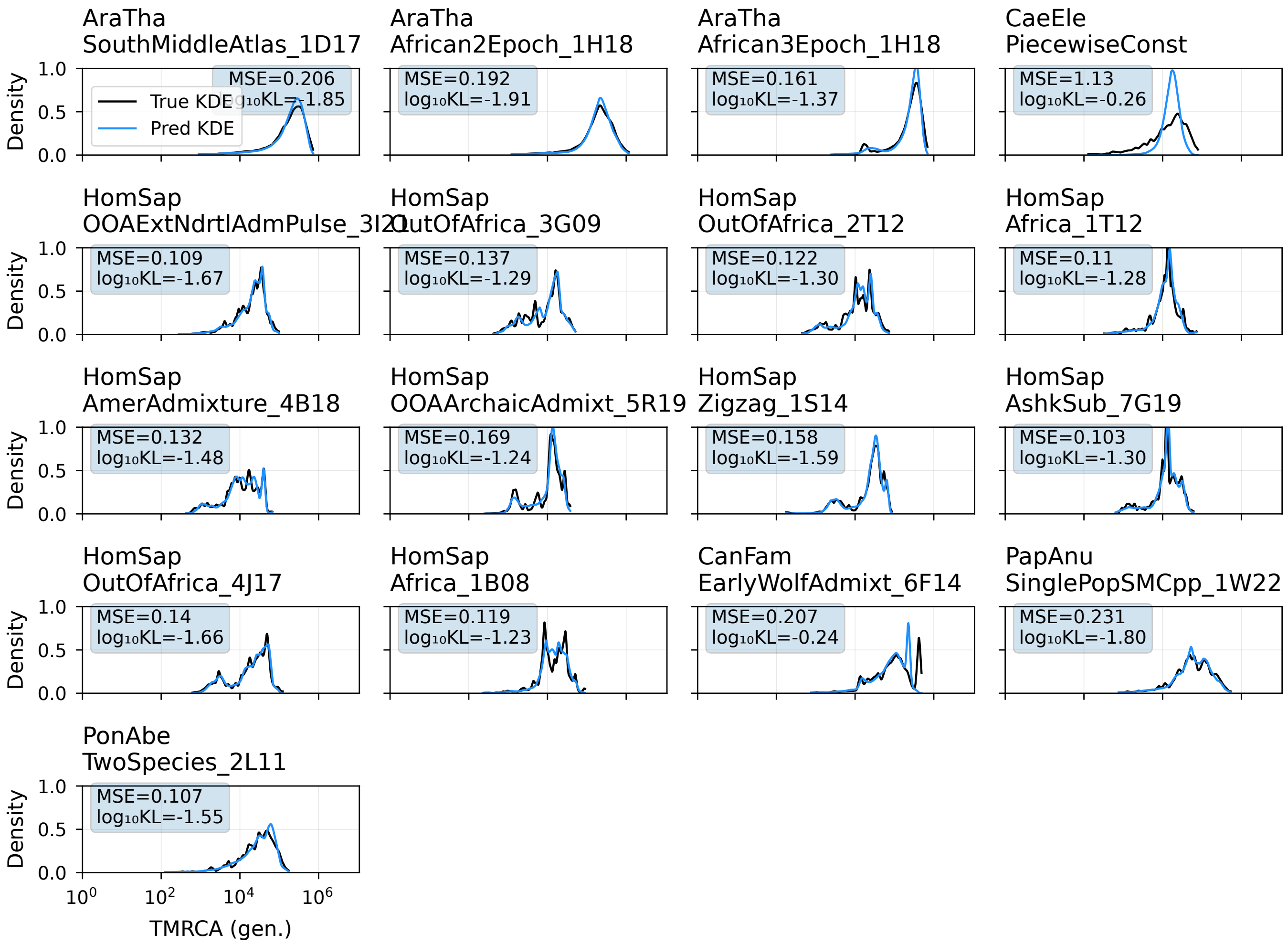

Fig. S8. Evaluation of inferred marginal coalescence distributions (dashed line) against the true distributions (shaded line), highlighting the model's **capacity** - its ability to distinguish scenarios based on context alone. The model generalizes to stdpopsim v0.2 simulations with varying mutation, recombination rates, and demography. Distributions are aggregated from pairwise inferences and visualized as kernel densities. All results are from new simulations, excluded from training. Unlike the preceding figure (Fig. S7), this one includes simulations with a genetic map.

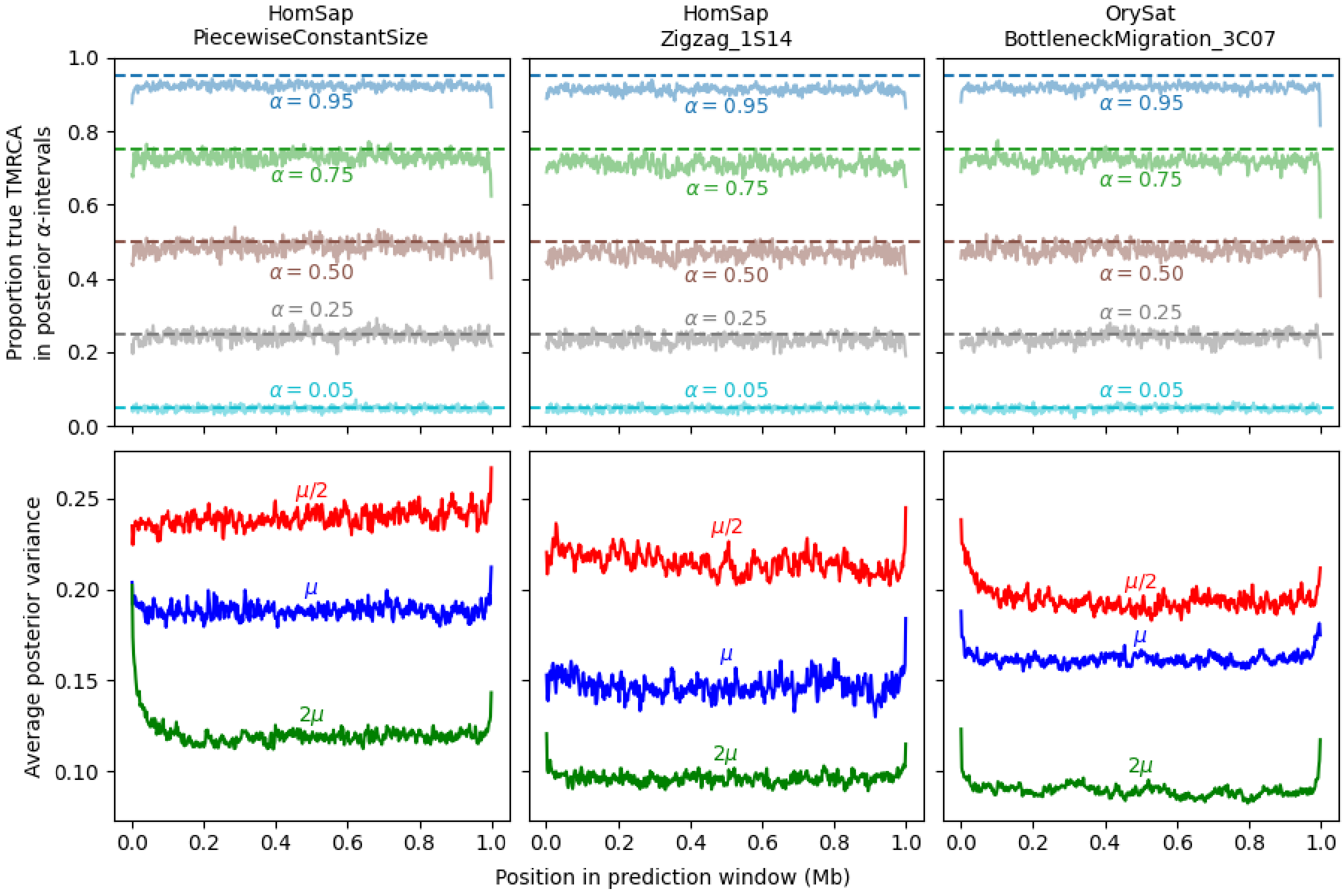

Fig. S9. The approximate posterior distributions sampled from by `cxt` give well-calibrated estimates of uncertainty for predicted TMRCAs, across a range of scenarios. Top row: the proportion of true TMRCAs that fall within credibility intervals of a given width (e.g. a given proportion of posterior mass), as a function of position in the 1Mb prediction frame. Credibility intervals are calculated from quantiles of 100 samples from the approximate posterior for each pivot pair, and the proportion covering the true TMRCAs are calculated over 1000 independent pivot pairs (e.g. from distinct genealogical simulations). The expected proportions (for an exact posterior) are shown as dashed lines. Empirically, the approximate posteriors are asymptotically well-calibrated, although slightly over-concentrated for larger interval widths (likely due to the time discretization used by `cxt`). The decrease in coverage for the last 2Kb window is due to a tendency of the LLM to introduce large jumps in TMRCA in this window. Bottom row: the posterior variance, averaged over 1000 independent pivot pairs, as a function of position in the prediction frame and mutation rate (where μ stands for the species' mutation rate in `stdpopsim`). As mutational information accrues, the posteriors become more precise, and lose precision towards the boundaries of the prediction frame where local context has been clipped.

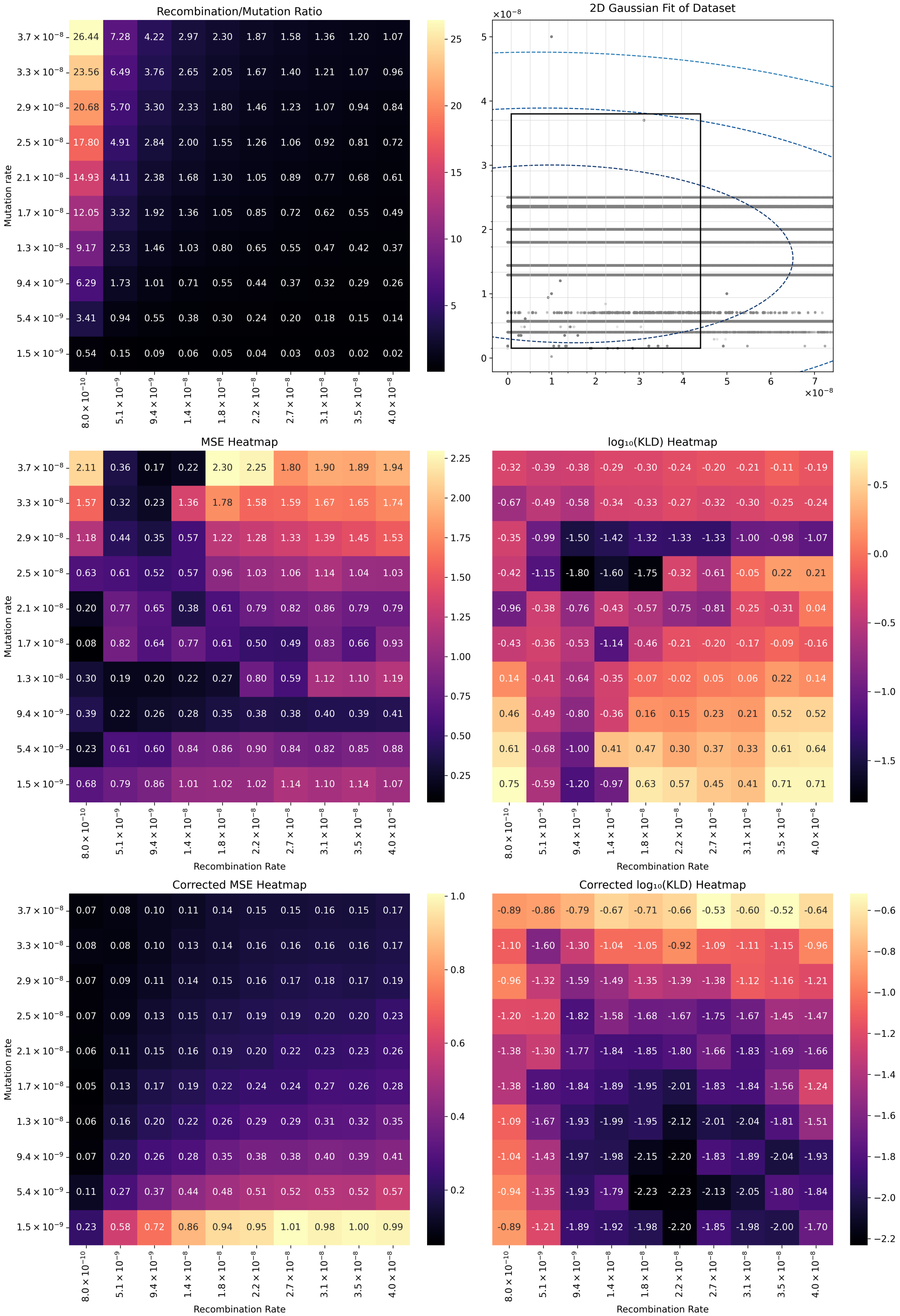

Fig. S10. Interpretation and limitations of `cxt` by testing various recombination to mutation ratios (top left). The ratios have been chosen in such a way that most of stdpopsim v0.2 simulations, with the exception of some outliers, fall into this rectangle (top right). The middle heatmaps show MSE and KLD values of `cxt` inferences (uncalibrated) over the entire grid, while the bottom heatmaps show mutation-rate-calibrated metrics. For four representative examples from the corners of this grid, see Figure S11.

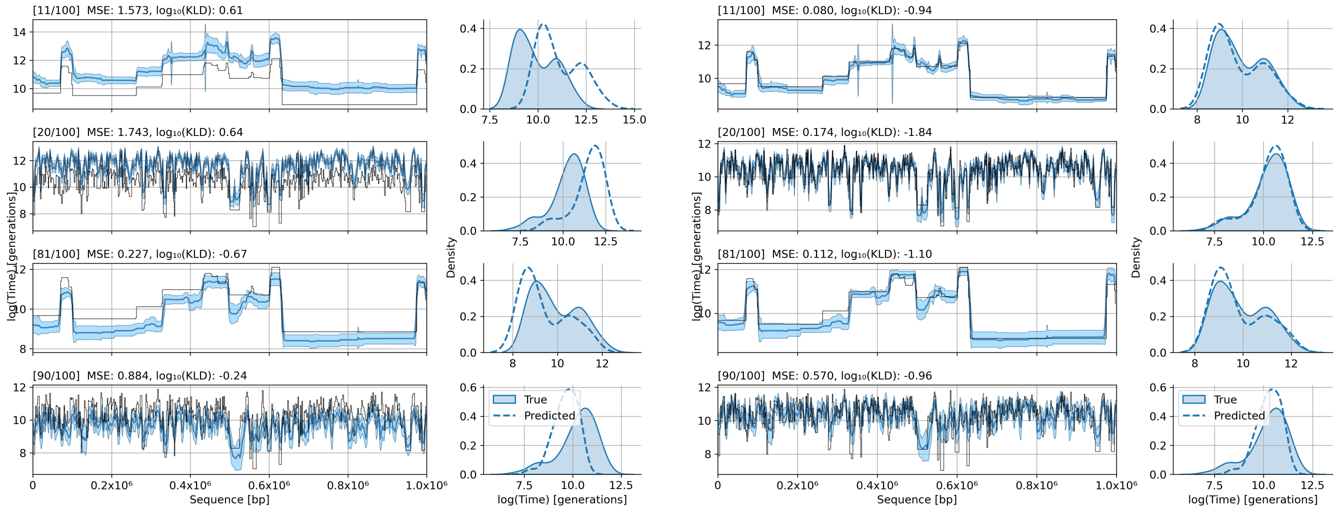

(a) Uncalibrated **(b)** Calibrated

Fig. S11. Interpretation and limitations of `cxt` zoomed into four representative cases from the corners of the interpolation grid (Figure S10), starting from top left to bottom right, with MSE and KLD indicated in the subtitles. These plots show the TMRCA along the sequence, while both right sides show the marginal distribution, true and inferred, respectively. The left four panels (**a**) are uncalibrated and the right four (**b**) after calibration.

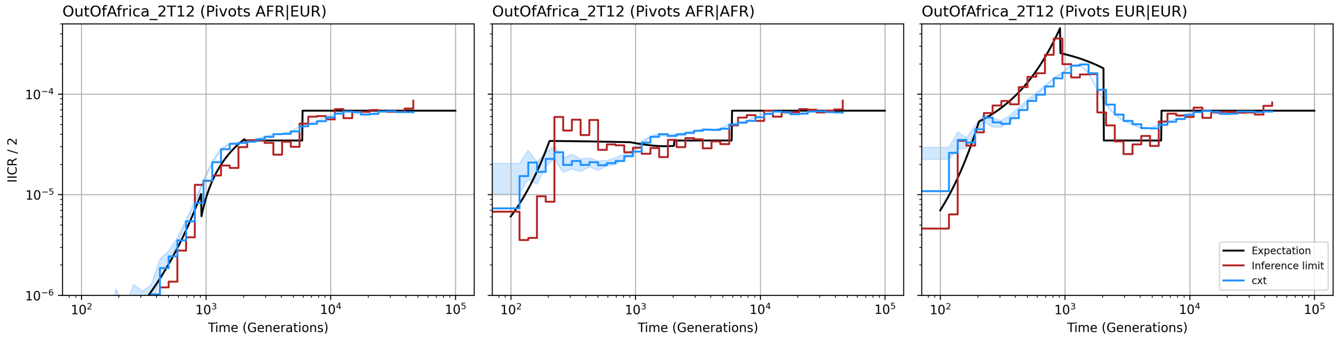

Fig. S12. Inverse-instantaneous coalescence rate calculation of a two population out-of-Africa demography for *H. sapiens* using (**left**) a mixture of African and European samples for cross-coalescence rate estimation, (**middle**) only African samples or (**right**) only European samples. The inference of pairwise-coalescence events leads to the implicit inference of demography estimates through the marginal coalescence distribution assuming coalescence occurs as a Poisson process (see methods). For each scenario 10 Mb and 25 diploid samples have been used to achieve resolution throughout the specified time-windows.

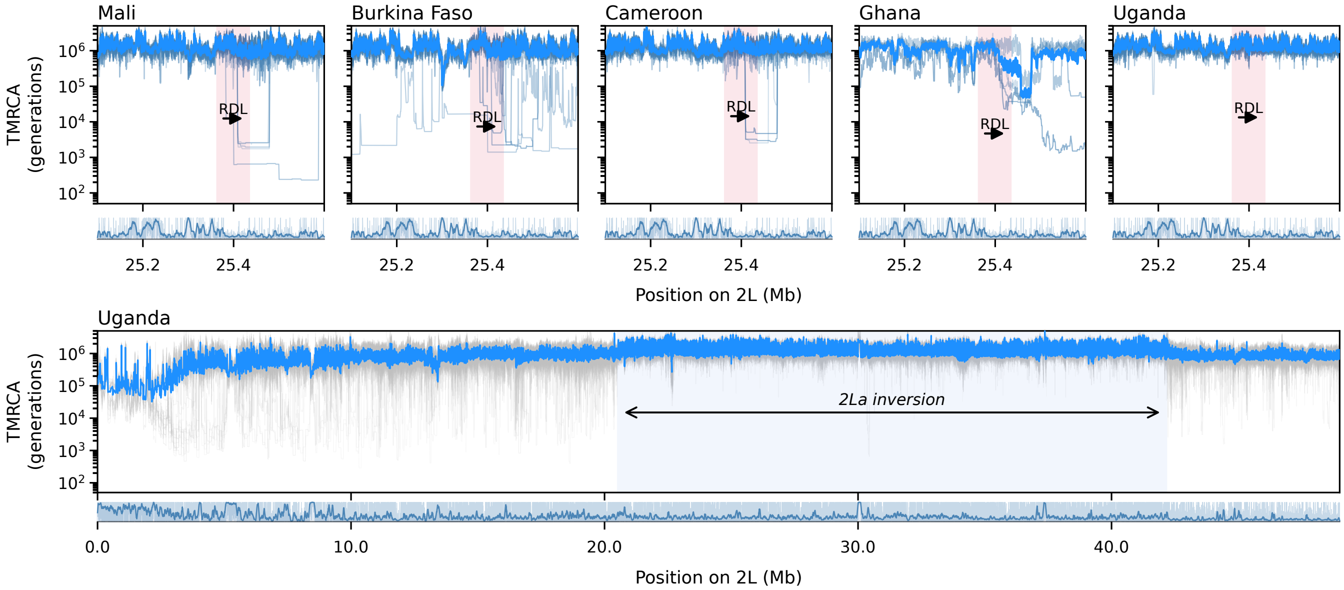

Fig. S13. Inference of coalescent-time landscapes in the Ag1000G *A. gambiae* dataset across five African populations using `cxt`. In the upper panel, we analyze the *Rdl* region for Burkina Faso, Mali, Cameroon, Ghana, and Uganda. For these countries, we infer coalescent times for 25 focal haplotype pairs per population; for Ghana, we analyze five diploid individuals. Light-blue curves indicate per-pair coalescent-time trajectories inferred by `cxt`, with the mean trend shown in darker blue. The lower panel shows genome-wide coalescent-time landscapes across the entirety of chromosome 2L for Uganda samples. Missing data density is shown beneath both the regional and genome-wide panels as a genome browser track.

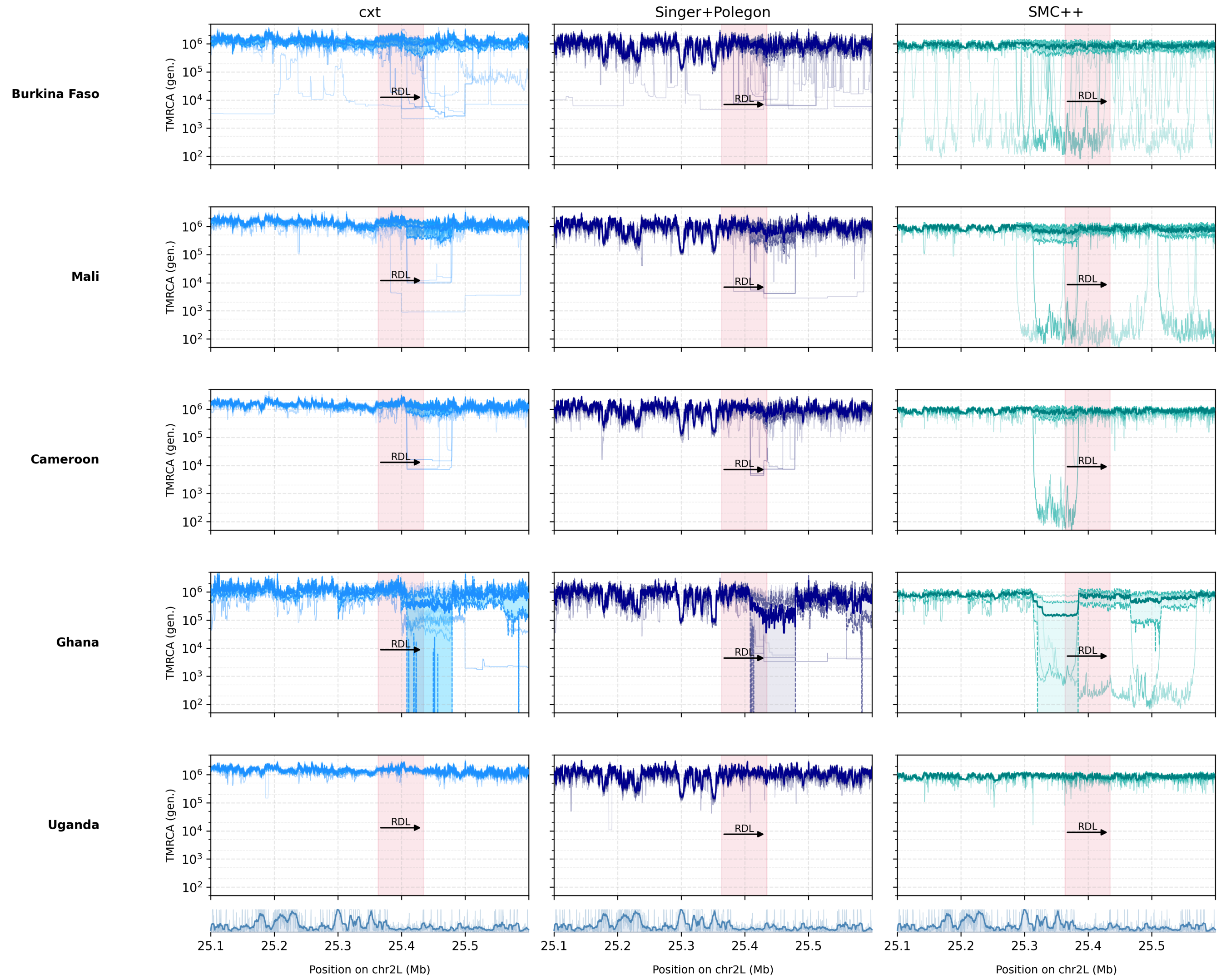

Fig. S14. Inference of coalescent-time landscapes in the Ag1000G *A. gambiae* dataset across five African populations (Upper panel). For Burkina Faso, Mali, Cameroon, and Uganda, we analyze 25 focal pairs per population; for Ghana, we analyzed only five diploid individuals. Panels show the *Rdl* region (highlighted in red), with estimates from `cxt` (left), `Singer+Polegon` (middle), and `SMC++` (right). Light-blue curves indicate per-pair inferences. The lower two panels show coalescent-time landscapes for the entirety of chr 2L for Uganda samples. Missing data density is shown beneath both the upper and lower panels as a genome browser track.

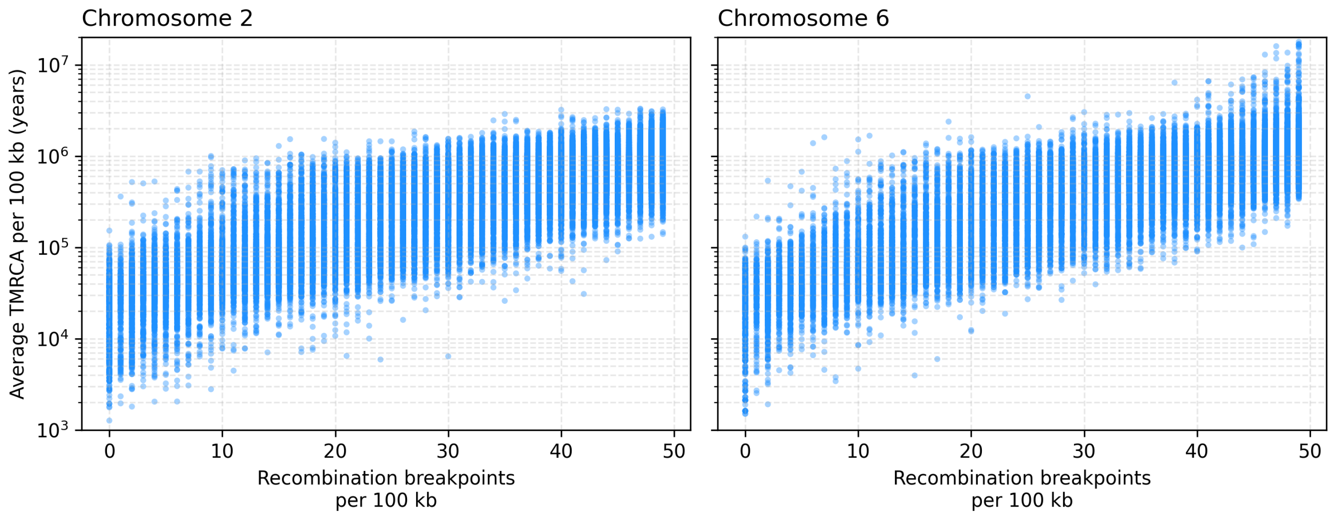

Fig. S15. Comparison of the average TMRCA (with generation time of 28 years) for adjacent 100 Kb windows and the average number of recombination breakpoints for 25 pivot pairs using the inferences from the 1000 Genomes Project for chromosome 2 (**left**) and chromosome 6 (**right**).
